# Comparative gene expression analysis reveals that multiple mechanisms regulate the weeping trait in *Prunus mume*

**DOI:** 10.1038/s41598-021-81892-3

**Published:** 2021-01-29

**Authors:** Lulu Li, Yichi Zhang, Tangchun Zheng, Xiaokang Zhuo, Ping Li, Like Qiu, Weichao Liu, Jia Wang, Tangren Cheng, Qixiang Zhang

**Affiliations:** Beijing Advanced Innovation Center for Tree Breeding By Molecular Design, Beijing Key Laboratory of Ornamental Plants Germplasm Innovation & Molecular Breeding, National Engineering Research Center for Floriculture, Beijing Laboratory of Urban and Rural Ecological Environment, Engineering Research Center of Landscape Environment of Ministry of Education, Key Laboratory of Genetics and Breeding in Forest Trees and Ornamental Plants of Ministry of Education, School of Landscape Architecture, Beijing Forestry University, Beijing, 100083 China

**Keywords:** Plant hormones, Plant molecular biology

## Abstract

*Prunus mume* (also known as Mei) is an important ornamental plant that is popular with Asians. The weeping trait in *P. mume* has attracted the attention of researchers for its high ornamental value. However, the formation of the weeping trait of woody plants is a complex process and the molecular basis of weeping stem development is unclear. Here, the morphological and histochemical characteristics and transcriptome profiles of upright and weeping stems from *P. mume* were studied. Significant alterations in the histochemical characteristics of upright and weeping stems were observed, and the absence of phloem fibres and less xylem in weeping stems might be responsible for their inability to resist gravity and to grow downward. Transcriptome analysis showed that differentially expressed genes (DEGs) were enriched in phenylpropanoid biosynthesis and phytohormone signal transduction pathways. To investigate the differential responses to hormones, upright and weeping stems were treated with IAA (auxin) and GA_3_ (gibberellin A3), respectively, and the results revealed that weeping stems had a weaker IAA response ability and reduced upward bending angles than upright stems. On the contrary, weeping stems had increased upward bending angles than upright stems with GA_3_ treatment. Compared to upright stems, interestingly, DEGs associated with diterpenoid biosynthesis and phenylpropanoid biosynthesis were significantly enriched after being treated with IAA, and expression levels of genes associated with phenylpropanoid biosynthesis, ABC transporters, glycosylphosphatidylinositol (GPI)—anchor biosynthesis were altered after being treated with GA_3_ in weeping stems. Those results reveal that multiple molecular mechanisms regulate the formation of weeping trait in *P. mume*, which lays a theoretical foundation for the cultivation of new varieties.

## Introduction

Plant architecture, which is closely associated with stem and bud development, is an important ornamental characteristic of woody plants. According to the direction of growth of the stem, ornamental woody plants can be divided into three types, straight-stem (stems grow upward), pendulous-stem (stems grow horizontally or downward) and tortuous-stem types (stems grow twisting naturally)^1^. Pendulous-stem (weeping) plants have high ornamental value during the leaf expansion period and after defoliation because of their naturally weeping stems and peculiar shape. Plants with the pendulous-branched (weeping) trait are observed in the herbaceous plant *Arabidopsis*^2^ and many woody species, such as willow (*Salix matsudana*)^3^, *Prunus persica*^4^, and *Morus alba*^5,6^. However, the phenotype of weeping traits is complicated. The direction of plant branch growth is to adapt to the stimulus of external environment, such as gravity, light, and mechanical forces. In response to these environmental stimuli, a large number of factors (genetic background, hormone, and nutrition, etc.) regulate cell division and growth leading to the formation of specific branch architecture^7^. Thus, the mechanism of the formation of weeping trait is diverse in different species. The weeping trait of willow is caused by the lack of mechanical support due to the excessive elongation of the stem^3^. Several studies have demonstrated that abnormal geotropic growth is involved in weeping phenotypes. The weeping trait in peach is caused by the mutation in *WEEP* gene which resulted in abnormal gravitropic perception^8^. Similarly, in *Arabidopsis sgr3-1* mutant, the lateral branches of the inflorescence stem grow horizontally or downwards. Mutation of the *SGR3* (*SHOOT GRAVITROPISM 3*) gene causes a defect in vacuole function or may interfere with amyloplast movement, resulting in a reduced ability to sense gravity^2^. *LAZY1* gene promotes narrow branch angel and weeping trait in multiple woody species, such as poplar^9^, birch^10^, and apple^11^ by regulating gravitropic response pathways. In addition, abnormal phototropic growth can also contribute to weeping trait in some species^9–11^. Studies of *Arabidopsis*, maize, and rice have shown that *LAZY* genes are involved in modulating gravitropism through regulating polar auxin transport^12–15^. Overexpression of *TAC1* (*TILLER ANGLE CONTROL 1*), another IGT family gene in *Arabidopsis*, plum, peach, and poplar, results in widening their branch angles in response to light and photosynthetic signals^9,16,17^. Moreover, phytohormones have vital roles in stem development and weeping trait formation. Transcriptome analysis of weeping and upright branches in willow showed that a large number of genes in hormone signal transduction, auxin and gibberellin (GA) biosynthesis pathways display differential expression and those genes may regulate the stem elongation and weeping trait^3^. In *P. persica* (peach), GA_3_ content increased from the base to the tip of a weeping branch where the GA content was higher than that at the tip of a standard branch, and the distribution of lignin was consistent with that of GA, indicating that the biosynthesis of lignin may be regulated by GA in peach. Additionally, an uneven distribution of GA in the adaxial and lower shoots results in uneven development of secondary xylem, leading to the weeping trait in *P. persica*^4^.

*Prunus mume*, a famous woody ornamental plant, is adopted in gardens as an important landscape plant due to its rich flower colors and branch types. *P. mume* ‘Fentai Chuizhi’ and *P. mume* ‘Liuban’ are weeping and upright varieties, respectively. And the branch growth models of weeping and upright branches based on the angle at multiple points on a branch have been established, and the models showed that there were significant differences in directions of branch growth during stem elongation stage between weeping and upright stems^18^. In addition, when the upright and weeping buds are grafted to the same rootstock at the same angle, two kinds of stem still grow in different directions^19^. Our previous studies have been performed to mine the molecular markers of weeping traits in *P. mume*. Quantitative trait locus (QTL) analysis of F_1_ generation of *P. mume* ‘Liuban’ × ‘Fentai Chuizhi’ showed that the weeping trait might be controlled by one major gene and several minor genes, and the major gene *pl* was located in the region of 10.54–11.68 Mb on chromosome 7^20^. Resequencing analysis of more than 330 varieties of *P. mume* showed that several candidate genes on chromosome 7 are related to the weeping trait^21^. However, the weeping trait in *P. mume* was complex and its molecular mechanism and regulatory networks remain to be investigated. In this study, we analyzed the transcriptome profiles and phytohormone response in the upright and weeping stems using RNA sequencing combined with morphological observation, by which to reveal the mechanism of the weeping trait as well as hormone control of the shoot architecture in *P. mume*.

## Results

### Morphological and histochemical characteristics of the weeping population

The F_1_ population of *P. mume* ‘Liuban’ (upright type) × ‘Fentai Chuizhi’ (weeping type) revealed an obvious separation of branch type characters^22^. The grafting progenies of F_1_ population that display upright and weeping trait were selected to observe the branch angle, respectively. Stem angles were observed 400 min on the distal side of the branch. As showed in Supplementary video 1, the deviation angles of upright branches changed rapidly at the range of 0–150 min and exhibited no significant changes since 150 min, while deviation angles of weeping stems significantly changed during 0–300 min and barely changed after 300 min (Supplementary video 1). Different contents (1 mg/L, 2 mg/L, 3 mg/L) of IAA and GA_3_ were applied on the adaxial side of two stem types to observe the angle changes after 400 min (Table S1). When the hormone concentration was 1 mg/L, neither of the branch deviation angle changes was obvious. It was abandoned, as the inconducive to the observation and measurement of angle changes. When the hormone concentration was 3 mg/L, angle changes of weeping stems coated with IAA were nearly 90°, which suggested that the excessive concentration of exogenous hormones might have a negative impact on the growth and development of the internal structure of the stem. Therefore, the angles of weeping and upright stems were measured after being treated with 2 mg/L IAA or GA_3_ for 6 h. The change in the deviation angle of the horizontal branch from the direction of gravity is positive ( +) and negative (−).

**Figure 1:**
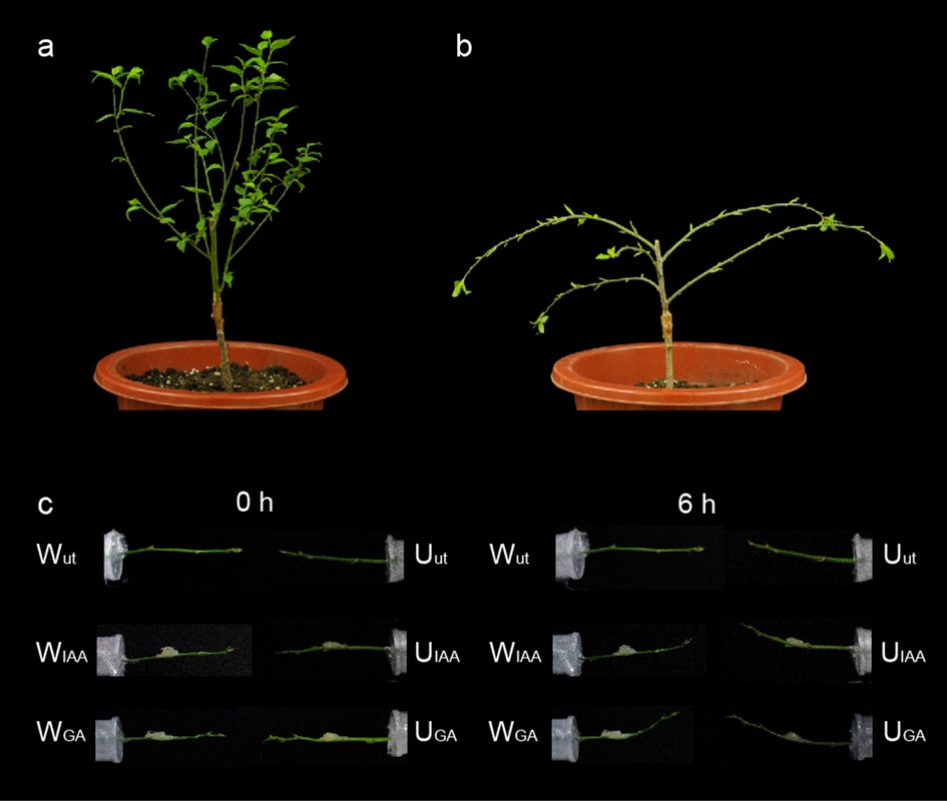
Morphological and hormone responsive characteristics of upright and weeping stems of *P. mume*. (**a**) Morphological characteristics of grafting progenies of F_1_ population with the upright trait; (**b**) Morphological characteristics of grafting progenies of F_1_ population with the weeping trait; (**c**) Changes in angles of upright and weeping stems in different treatments; U_ut_, upright stem; W_ut_, weeping stem; U_IAA_, upright stem with IAA treatment; W_IAA_, weeping stem with IAA treatment; U_GA_, upright stem with GA_3_ treatment; W_GA_, weeping stem with GA_3_ treatment.

As showed in Table 1 and Fig. 1, the angle of straight and pendulous stems differed significantly after 6 h in different treatments. After laying both stems horizontally for 6 h, both upright and weeping stems could grow by bending upward slightly, which is the direction of the light source. However, the angle of upright stem (( +) 2.92 ± 0.11) was significantly larger than that of weeping stem (( +) 0.91 ± 0.07) (Table 1, Fig. 1c). The angle of upright stem with IAA treatment was significantly greater than that of upright stem. On the contrary, deviation angle of upright stem was significantly smaller than that of upright stem after GA_3_ treatment.

**Table 1:** Differences between upright and weeping stems in response to 2 mg/L IAA and GA_3_ treatments.

	Upright stem (°)	Weeping stem (°)	Weeping/Upright Ratio (%)
CK	( +) 2.92 ± 0.11^a^	( +) 0.91 ± 0.07^b^	31.2
IAA	( +) 10.73 ± 0.25^a^	( +) 1.65 ± 0.17^b^	15.4
GA_3_	( +) 9.00 ± 0.29^b^	( +) 13.99 ± 0.31^a^	155.4

The data showed the deviation angle of the stem from the horizontal direction, ( +) represents bending in a negative gravity direction. Different letters indicate a significant difference (*P* < 0.05) based on one-way ANOVA. Error bars represent one standard error of the mean (n = 42).

**Figure 2:**
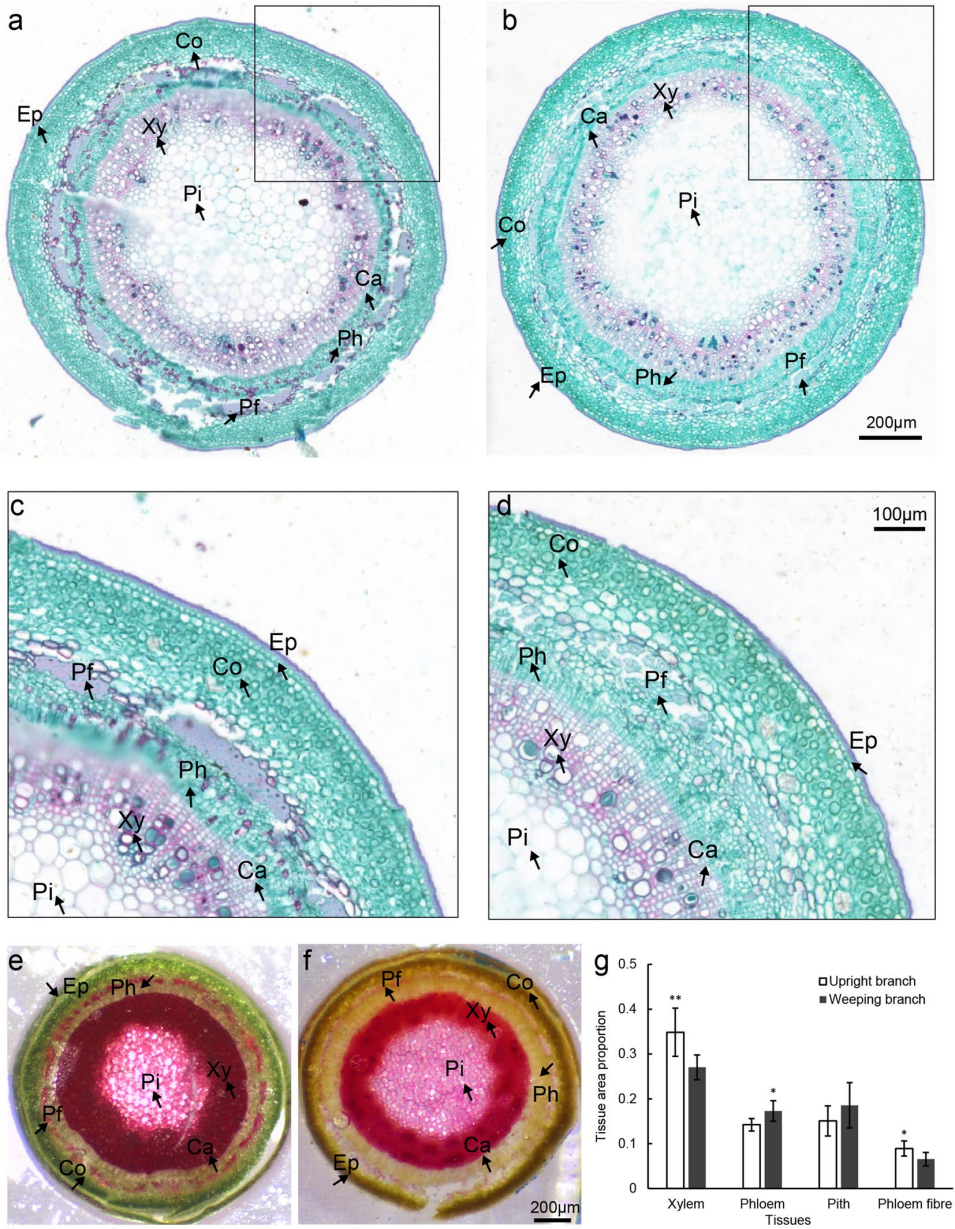
Histochemical characteristics of upright and weeping stems of *P. mume*. (**a**) Cross-section of elongating annual stems with the upright trait; (**b**) Cross-section of elongating annual shoots with the weeping trait; (**c**) Enlargement of the section in the black box of **a**; (**d**) Enlargement of the section in the black box of **b**; (**e**) Phloroglucinol-HCL staining of the middle segment of upright stem; (**f**) Phloroglucinol-HCL staining of the middle segment of weep stem; (**g**) The area ratios of xylem, phloem, pith, and phloem fibre tissues to cross-sectional area in the middle of elongating annual upright and weeping stems. Single and double asterisks represent *P* < 0.05 and *P* < 0.01, respectively. Error bars represent one standard error of the mean (n = 3). Ep, epidermal cell; Co, cortex; Ph, phloem; Pf, phloem fibre; Xy, xylem; Pi, pith.

Paraffin sectioning and safranin green staining were conducted to further reveal the differences in histological structures between upright and weeping stems. As showed in Fig. 2a,b, the cross-sections of upright and weeping stems were both circular. Compared to upright stem, the xylem area and phloem fibre area of weeping stem have small proportions, while the phloem area accounts for a large proportion (Fig. 2c,d,g). In annual upright stem, the phloem fibre cells in the fibre bundle are arranged neatly and have thicker lignified cell wall that were stained red with saffron (Fig. 2c), while the phloem fibre cells were disorganized with different shapes and sizes, and some cells have no lignified cell wall in weeping stem (Fig. 2d). Phloroglucinol—HCl staining analysis yielded similar results (Fig. 2e,f,g).

### Transcriptomic data in upright and weeping stems

RNA samples from untreated upright (U_ut_) and weeping stem (W_ut_) and samples from weeping and upright stem after 6 h of water (W_mock_ and U_mock_), IAA (U_IAA_ and W_IAA_) or GA_3_ (U_GA_ and W_GA_) treatment were extracted to generate cDNA libraries, respectively. Through transcriptome sequencing analysis, a total of 81.48 million clean reads were generated. The effective data of each library was more than 6.24 Gb, and the Q30 base percentage was 95%, indicating that the sequencing quality was reliable (Table S2). More than 79% of the highly quality reads from individual samples could be mapped on the genome of *P. mume*. A total of 19, 512 genes were identified accounting 92.83% of all reference genes (21, 019). And more than 93% of genes were already known and about 6% of genes were new (Table S3). More details of RNA-seq reads, genome alignment, and gene number are shown in Table S2 and Table S3. All the raw read data were deposited in the Genome Sequence Archive under project ID PRJCA001723.

### Differentially expressed genes in upright and weeping stems of *P. mume*

We analysed unigene expression in eight libraries (U_ut_, W_ut_, W_mock_, U_mock_, U_IAA_, W_IAA_, U_GA_, and W_GA_) and normalized the values using fragments per kilobase million (FPKM). In order to investigate the influences of 6 h of horizontal placement on stems, we sampled after placing weeping and upright stems horizontally and treated them with water for 6 h as W_mock_ and U_mock_, respectively. A total of 86 DEGs were identified in W_mock_ vs. U_mock_ (Table S4). Venn diagram analysis showed that W_ut_ vs. U_ut_ and W_mock_ vs. U_mock_ shared 121 DEGs, and 365 DEGs existed specifically in W_mock_ vs. U_mock_ (Fig. S1a). KEGG analysis of 365 DEGs that existed specifically in W_mock_ vs. U_mock_ indicated that genes in glutathione metabolism (ko00480), metabolic pathways (ko01100), and linoleic acid metabolism (ko00591) pathways were significantly enriched, suggesting that the upright and weeping stems were different in response to in vitro culture conditions (Fig. S1a, Fig. S2a).

There were 317 DEGs in U_ut_ vs. W_ut_, represented by the differences between upright and weeping stems (Table S5). W_IAA_ vs. U_IAA_ and W_GA_ vs. U_GA_ represented the differences in response to IAA and GA_3_ between upright and weeping stems, respectively. There were 896 and 1, 312 DEGs in W_IAA_ vs. U_IAA_ and W_GA_ vs. U_GA_, respectively (Fig. S1a,b, Tables S6, and S7). The clustered expression patterns of all DEGs between upright and weeping stems upon different treatments were created based on their log2 expression level values (FPKM) using STEM software^23^. Expression trend analysis split the DEGs in the three comparisons (W_ut_ vs. U_ut_, W_IAA_ vs. U_IAA_, W_GA_ vs. U_GA_) into 20 clusters: profile 0 – profile 19 with distinct expression patterns (Fig. S1c,d; Table S8). There are six trend models (profile 2/15/4/19/0/18) that changed significantly (*p* < 0.05). Genes in profile 2 (237) and profile 4 (160) were down-regulated in W_ut_, while genes in profile 15 (257), profile 19 (167), profile 0 (167), and profile 18 (148) were up-regulated in W_ut_ compared to U_ut_, showing that those genes may involve in regulating the formation of branch type in *P. mume*. Genes in profile 15 showed high transcript levels in W_ut_, W_IAA_, and W_GA_, indicating that those genes were up-regulated in weeping stem in response to control, IAA or GA_3_ treatment. In contrast to profile 15, profile 4 included genes that were down-regulated in W_ut_, W_IAA_, and W_GA_. Genes in profile 4 and profile 15 might participate in the branch architecture regulated by IAA and GA_3_ in *P. mume*.

### GO and KEGG analysis of DEGs in upright and weeping stems

**Figure 3:**
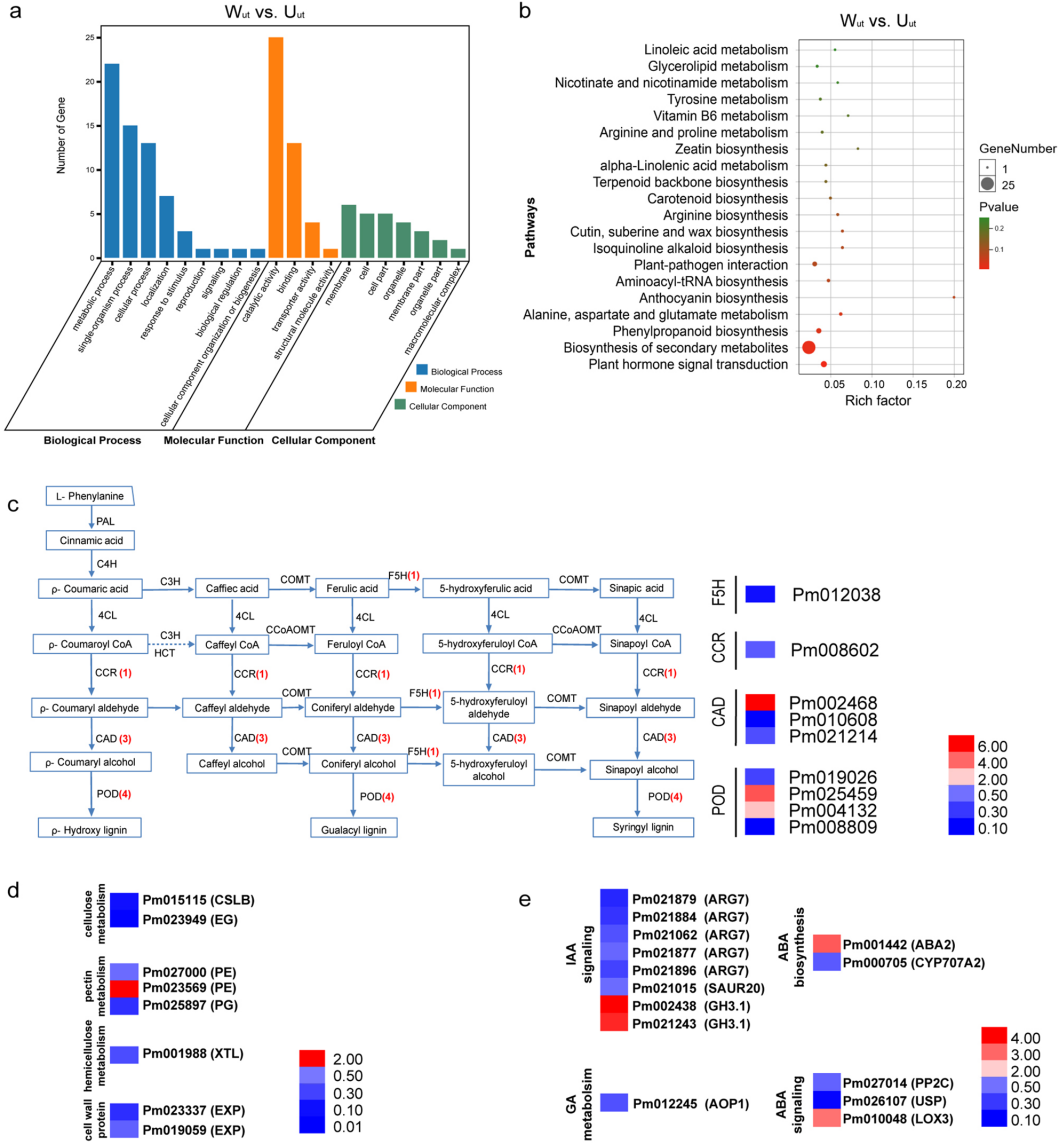
GO, KEGG cluster and enrichment pathway analysis. **(a)** GO category enrichment of DEGs in W_ut_ vs. U_ut_. (**b**) KEGG category enrichment of DEGs in W_ut_ vs. U_ut_; The y-axis indicates the pathway, and the x-axis indicates the enrichment factors corresponding to the pathway. The dot size represents the number of DEGs in the pathway, and the dot colour represents the *P*-value. **(c)** Clustering of DEGs involved in phenylpropanoid biosynthesis in upright and weeping stems of *P. mume*. *F5H*, *ferulate 5-hydroxylase*; *4CL*, *4 coumarate CoA ligase*; *HCT*, *hydroxycinnamoyl transferase*; *CCoAOMT*, *Caffeoyl-CoA O-methytransferase*; *CCR*, *Cinnamoyl CoA reductase*; *CAD*, *cinnamyl alcohol dehydrogenase*; *PLR*, *pinoresinol-lariciresinol reductase*; *POD*, *peroxidase*. The red numbers represent the number of DEGs. **(d)** Clustering of DEGs involved in plant cell wall formation in upright and weeping stems of *P. mume*. *CSLB*, *cellulose synthase-like B*; *EG*, *cellulose synthase-like*; *PE*, *pectinesterase*; *PG*, *polygalacturonase*; *XTL*, *xyloglucan galactosyltransferase*; *EXP*, *expensin*; **(e)** Clustering of DEGs involved in phytohormones in upright and weeping stems of *P. mume*. *ARG*, *indole-3-acetic acid-induced protein*; *SAUR, auxin-responsive protein*; *GH3.1*, *indole-3-acetic acid-amido synthetase*; *AOP1*, *2-oxoglutarate-dependent dioxygenase AOP1*; *ABA2*, *ABA deficient 2*; *CYP707A2*, *cytochrome P450, family 707, subfamily A, polypeptide 2*; *PP2C*, *Protein phosphatase 2C*; *USP*, *universal stress protein*; *LOX*, *lipoxygenase*. Red and blue indicate up- and down-regulated genes, respectively (fold change).

To examine putative functional differences between upright and weeping stems, we conducted GO, KEGG, and MapMan annotation with 316 DEGs from W_ut_ vs. U_ut_. DEGs were mainly divided into three GO categories: biological processes, cell components and molecular functions (Fig. 3a). Metabolic process (GO: 0008152), single-organism process (GO: 0044699), and cellular process (GO: 0009987) were the most highly represented groups in the biological process category. Within the cellular component category, DEGs that corresponded to membrane (GO: 0016020) were the most abundant and catalytic activity (GO: 0003824) and binding (GO: 0005488) were the most abundant classes in the molecular function category. We further identified enriched GO terms in three categories that were over-represented (*P* < 0.05) in DEGs of W_ut_ vs. U_ut_, the results are shown in Table S9. Many genes involved in protein phosphorylation (GO: 0006468) and phosphorylation (GO: 0016310) have obvious differences in biological processes, and DEGs involved in hydrolase activity, acting on glycosyl bonds (GO: 0016798), alpha-1,4-glucosidase activity (GO: 0004558), and alpha-glucosidase activity (GO: 0090599) were enriched in molecular functions, suggesting that there might be differences in carbohydrate metabolism between upright and weeping stems. The DEGs in W_ut_ vs. U_ut_ were then subjected to KEGG pathway mapping, and the top 20 enriched pathways are shown in Fig. 3b. KEGG annotations showed that the pathways of plant hormone signal transduction (ko04075), biosynthesis of secondary metabolites (ko01110) and phenylpropanoid biosynthesis (ko00940) were enriched in W_ut_ vs. U_ut_, indicating that weeping and upright stems are different in hormone sensitivity and phenylpropanoid biosynthesis (Table S10). MapMan bins of “Metabolism_overview” showed similar results (Fig. S3a).

### Cluster analysis of genes involved in phenylpropanoid biosynthesis, cell wall biosynthesis, and phytohormone signaling

The putative functional homologues of nine genes encoding enzymes involved in phenylpropanoid biosynthesis were recognized, and their expression patterns in four tissues are shown in Fig. 3c. Three genes involved in phenylpropanoid biosynthesis were up-regulated, including *Pm002468* (*CAD, CINNAMYL ALCOHOL DEHYDROGENASE*), *Pm025459* (*POD, peroxidase*), and *Pm004132* (*POD*), and six genes were down-regulated (*Pm012038* (*F5H, ferulate 5-hydroxylase*), *Pm008602* (*CCR1, Cinnamoyl CoA reductase 1*), *Pm010608* (*CAD*), *Pm021214* (*CAD*), *Pm019026* (*POD*), and *Pm008809* (*POD*)) in W_ut_ compared to U_ut_.

Plant cell walls are composed of cellulose, hemicellulose, pectin, xylan, and cell wall proteins. Additionally, numerous genes related to cellulose, hemicellulose, pectin, and lignin biosynthesis were differentially expressed in W_ut_ vs. U_ut_. A total of eight DEGs were identified to be involved in cell wall in W_ut_ vs. U_ut_ (Fig. 3d). The *CSL* (*cellulose synthase-like*) gene encoding cellulose synthase-like proteins is an important gene related to cellulose biosynthesis in the cell wall. *CSL* gene (*Pm015115*) was down-regulated in weeping stem. *Pm023949* (*cellulose synthase-like*, *EG*) gene associated with cellulose degradation was also down-regulated in W_ut_. Instead of *Pm023569* (*PE*, *pectinesterase*), other genes encoding pectin degradation-related proteins (*Pm027000* and *Pm025897*), and *expansin* proteins (Pm019059 and Pm023337) were both down-regulated in weeping stem (Fig. 3d). These results suggested significant differences among plant cell wall biosynthesis and degradation between upright and weeping stems.

Compared with upright stem, 14 genes involved in phytohormone metabolism and signal transduction were differentially expressed (Fig. 3e). Among them, eight DEGs were related to IAA signal transduction, *ARG7s* (*Pm021879, Pm021884, Pm021062, Pm021877, Pm021896*) and *SAUR20* (*Pm021015*) were down-regulated while two *GH3.1s* (*Pm002438*, *Pm021243*) were up-regulated in weeping stems. In addition, GA metabolism gene *Pm012245* (*AOP1*) were down-regulated in weeping stems, two ABA biosynthesis genes (*Pm001442*, *Pm00705*) and three ABA signaling genes (*Pm027014*, *Pm026107*, *Pm010048*) were altered in weeping stems.

### Cluster analysis of differentially expressed transcription factors

**Figure 4:**
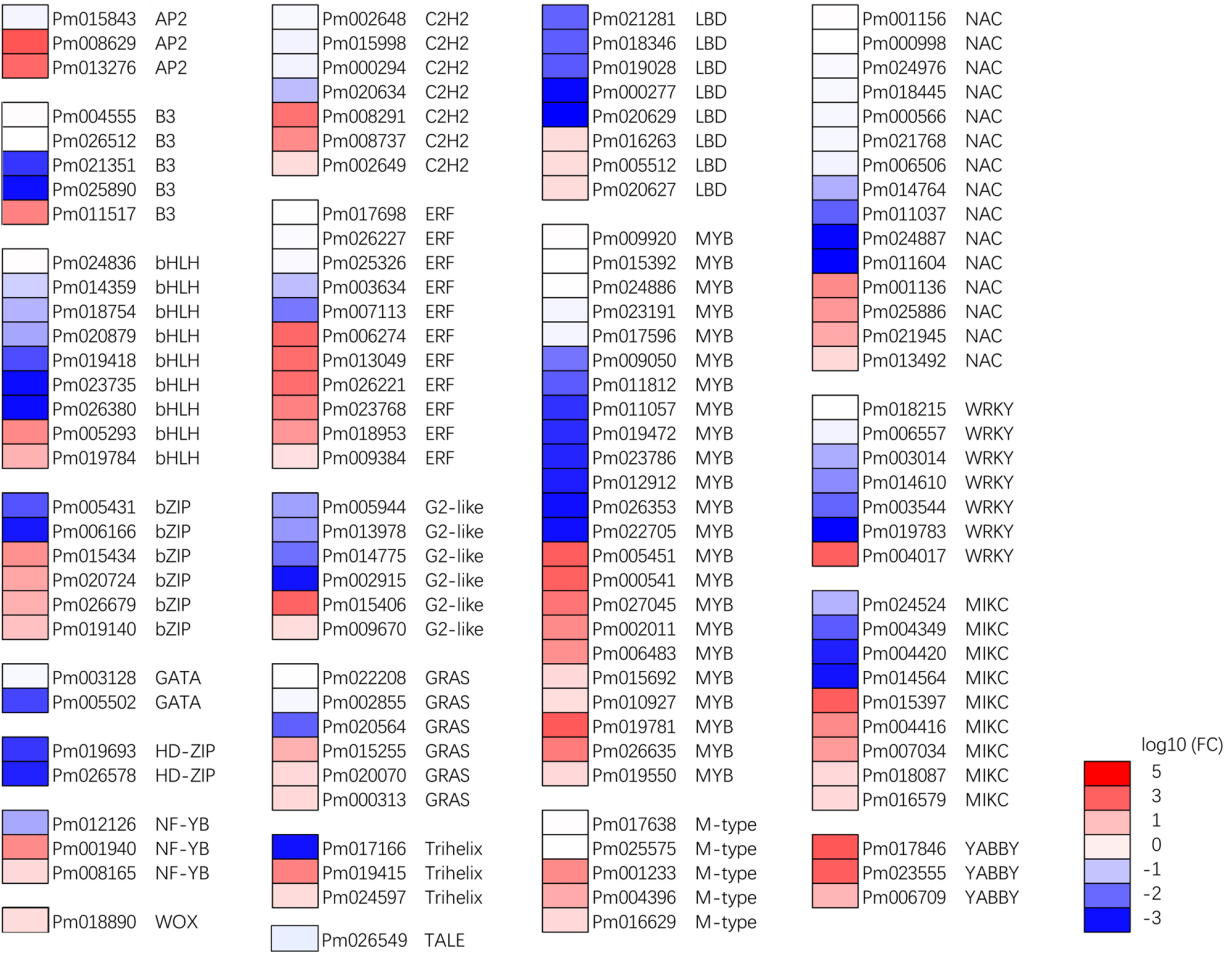
Clustering of differentially expressed transcription factors in upright and weeping stems of *P. mume*. Red and blue indicate up- and down-regulated genes, respectively, in the three comparisons (log10-fold change).

Transcription factors (TFs) play diverse roles in regulating the activity of many metabolic pathways during plant growth and development. A total of 135 TFs were differentially expressed in W_ut_ vs. U_ut_ (Table S11), which represented putative regulators of the weeping trait (57 up-regulated and 78 down-regulated in W_ut_) (Fig. 4). Among the differentially expressed TFs, the largest 11 groups of TFs with differential expression were MYB (23), NAC (15), ERF (11), bHLH (9), MIKC (9), LBD (8), WRKY (7), C2H2 (7), and C2-like (6), bZIP (6), and GRAS (6).

### Differences in response to IAA and GA_3_ treatments

In order to investigate the influences of 6 h of horizontal placement and water treatment on stems, we sampled after placing weeping and upright stems horizontally and treated them with water for 6 h as W_mock_ and U_mock_, respectively. W_IAA_ vs. W_mock_ and U_IAA_ vs. U_mock_ reflected the response of weeping and upright stems to auxin (Fig. S2b, c). Ribosome, plant hormone signal transduction, ribosome biosynthesis in eukaryotes, and biosynthesis of secondary metabolites were the four pathways with the most significant enrichment in both W_IAA_ vs. W_mock_ and U_IAA_ vs. U_mock_. It is worth noting that several GA biosynthesis genes, such as *GA20OX3*, *GA3OX1*, *KAO1*, were up-regulated in both W_IAA_ vs. W_mock_ and U_IAA_ vs. U_mock_, suggesting that IAA treatment might contribute to the GA synthesis in *P. mume* (Fig. S2 b, c, Tables S12–15). However, genes related to ABC transporters were enriched in U_IAA_ vs. U_mock_ but not in W_IAA_ vs. W_mock_, and phenylpropamoid biosynthesis genes were enriched in W_IAA_ vs. W_mock_ but not in U_IAA_ vs. U_mock_ (Fig. S2 b, c, Table S12-15). Similarly, W_GA_ vs. W_mock_ and U_GA_ vs. U_mock_ reflected the response of weeping and upright stems to gibberellin (Fig. S2d, e, Table S16-19). ABC transporter biosynthesis genes were significantly enriched in U_GA_ vs. U_mock_ rather than in W_GA_ vs. W_mock_. Those differences were further reflected in comparison of W_IAA_ vs. U_IAA_ and W_GA_ vs. U_GA_.

**Figure 5:**
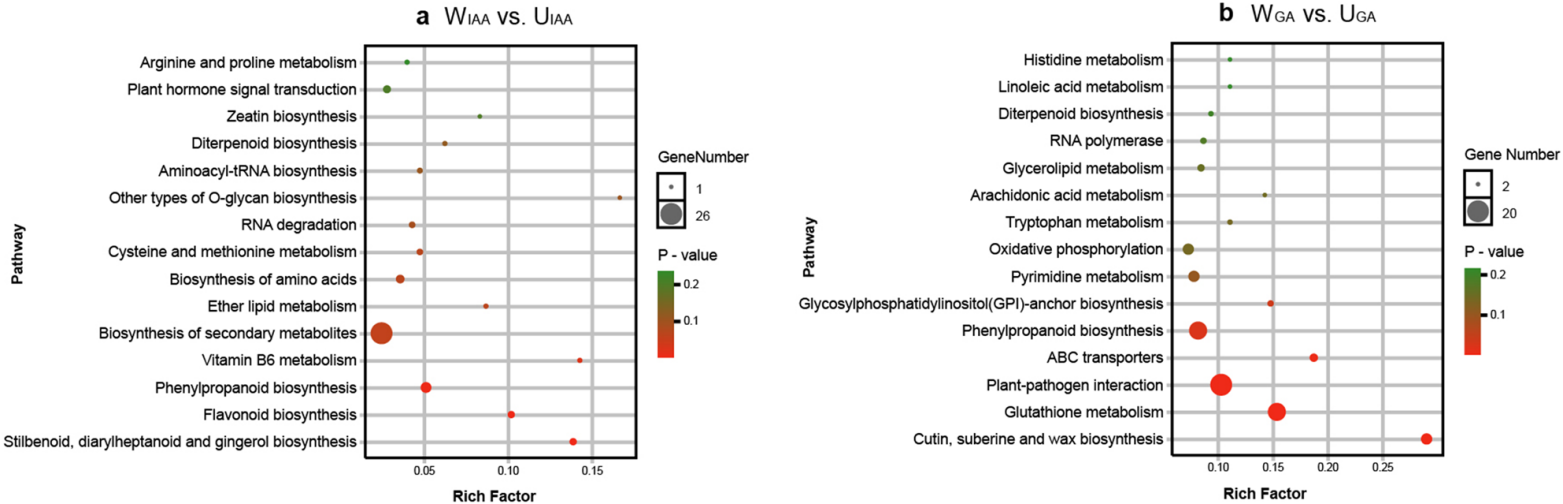
KEGG analyses reveal the differences in response of upright and weeping stems to IAA and GA treatments. (**a**) KEGG category enrichment of DEGs in W_IAA_ vs. U_IAA_. (**b**) KEGG category enrichment of DEGs in W_GA_ vs. U_GA_. The y-axis indicates the pathway, and the x-axis indicates the enrichment factors corresponding to the pathway. The dot size represents the number of DEGs in the pathway, and the dot colour represents the *P*-value.

**Figure 6:**
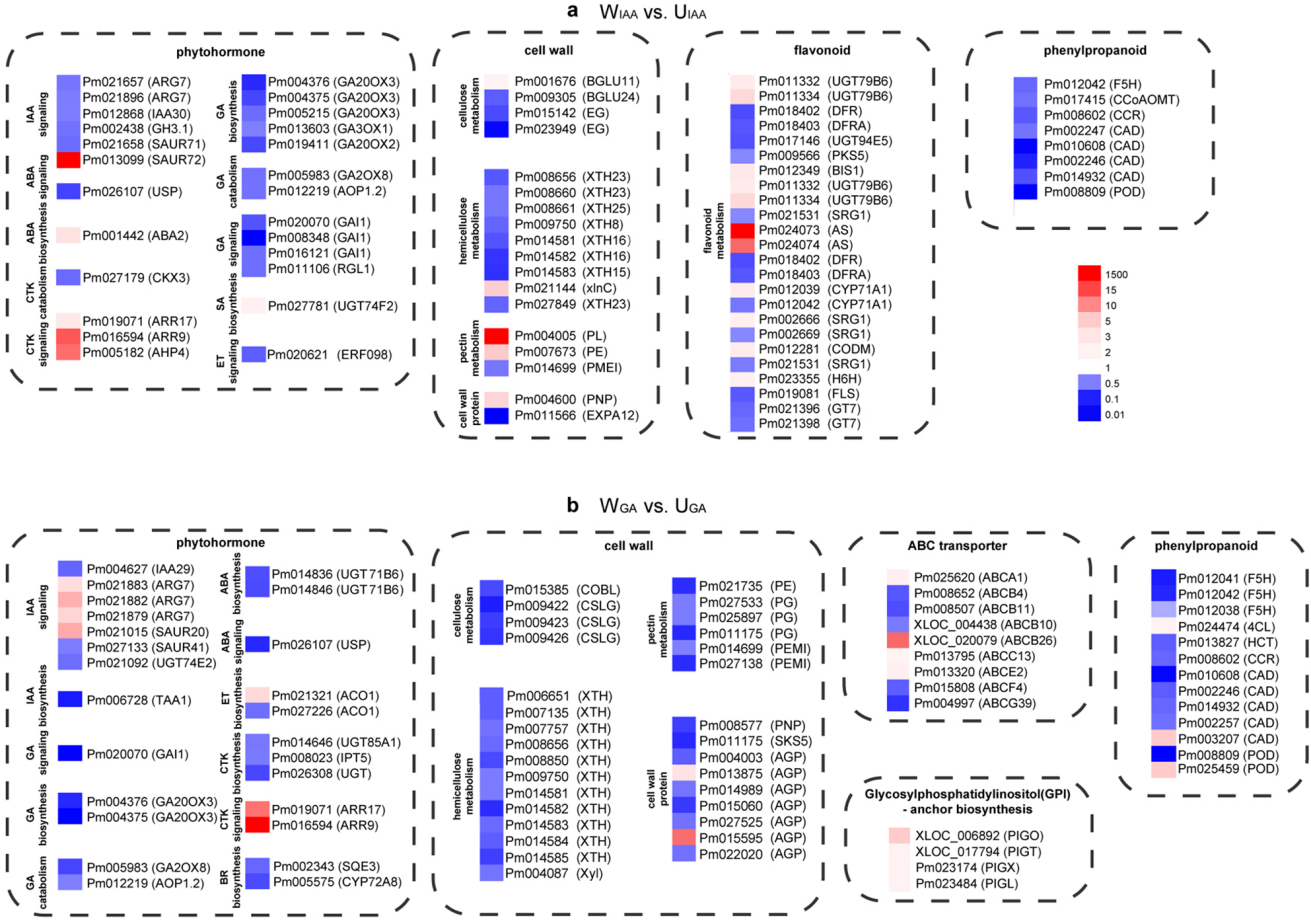
Clustering of DEGs in several pathways enriched in W_IAA_ vs. U_IAA_ and W_GA_ vs. U_GA_. (**a**) DEGs involved in phytohormones, cell wall, flavonoid, and phenylpropanoid metabolism in W_IAA_ vs. U_IAA_. (**b**) DEGs involved in phytohormones, cell wall, ABC transporter, Glycosylphophatidylinositol (GPI)—anchor, phenylpropanoid metabolism in W_GA_ vs. U_GA_. Red and blue indicate up- and down-regulated genes, respectively (fold change). *ARG*, *indole-3-acetic acid-induced protein*; *GH3.1*, *indole-3-acetic acid-amido synthetase*; *SAUR*, *auxin-responsive protein*; *UGT74E2*, *uridine diphosphate glycosyltransferase 74E2*; *TAA1, L-trytophan-pyruvate aminotransferase*; *GA20OX3*, *gibberellin 20 oxidase 2*; *GA3OX1*, *gibberellin 3 oxidase 1*; *GA2OX8*, gibberellin 2-beta-dioxygenase 8; *AOP1*, *inactive 2-oxoglutarate-dependent dioxygenase AOP2*; *GAI*, *gibberellic acid insensitive; RGL*, *RGA (repressor of GA)-like*; *USP, universal stress protein: ABA2*, *ABA deficient 2*; *UGT71B6*, *UDP-glucosyl transferase 71B6*; *CKX3*, *cytokinin oxidase 3*; *UGT85A1*, *uridine diphosphate glycosyltransferase 85A1*; *UGT*, *UDP-glucosyl transferase*; *IPT5*, *adenylate isopentenyltransferase 5*; *ARR*, *response regulator*; *AHP*: *histidine-containing phosphotransfer 4*; *UGT74F2*, *UDP-glucosyltransferase F2*; *ERF098*, *ethylene-responsive transcription factor 098*; *ACO1*, *ACC oxidase 1*; *SQE3*, *squalene epoxidase 3*; *CYP72A8*, *cytochrome P450*, *family 72*, *subfamily A*, *polypeptide 8*; *BGLU*, *β-glucosidase*; *EG*, *cellulose synthase-like*; *COBL*, *COBRA-like protein*; *CSLG*, *cellulose synthase-like G*; *XTH*, *xyloglucan endotransglucosylase*; *Xln*, *endo-1,4-beta-xylanase*; *Xyl*, *beta-xylosidase*; *PL*, *pectate lyase*; *PE*, *pectinesterase*; *PG*, *polygalacturonase*; *PMEI*, *pectinesterase inhibitor*; *PNP*, *plant natriuretic peptide*; *EXP*, *expansin*; *AGP*, *arabinogalactan protein*; *ERG*, *glycine-rich cell wall structural protein*; *SKS*, *SKU5 similar 5*; *UGT79B6*, *UDP-glycosyltransferase 79B6-like*; *DFR*, *dihydroflavonol-4-reductase-like*; *DFRA*, *anthocyanidin reductase-like*; *UGT94E5*, *beta-D-glucosyl crocetin beta-1,6-glucosyltransferase-like*; *PKS5*, *polyketide synthase 5-like*; *BIS1*, *3,5-dihydroxybiphenyl synthase-like*; *SRG1*, *protein DMR6-LIKE OXYGENASE 2-like*; *AS*, *hydroquinone glucosyltransferase-like*; *CYP71A1*, *cytochrome P450 CYP736A12-like*; *SRG1*, *senescence-related gene 1*; *CODM*, *codeine O-demethylase-like*; *H6H*, *protein DOWNY MILDEW RESISTANCE 6-like*; *FLS*, *flavonol synthase/flavanone 3-hydroxylase-like*; *GT7*, *UDP-glucose flavonoid 3-O-glucosyltransferase 7-like*; *F5H*, *ferulate 5-hydroxylase*; *CCoAOMT*, *Caffeoyl-CoA O-methytransferase*; *CCR*, *Cinnamoyl CoA reductase*; *CAD*, *cinnamyl alcohol dehydrogenase*; *POD*, *peroxidase*; *4CL*, *4 coumarate CoA ligase*; *HCT*, *hydroxycinnamoyl transferase*; *ABC*: *ATP Binding Cassette transporter*; *PIG*, *phosphatidylinositol-glycan biosynthesis protein*. Red and blue indicate up- and down-regulated genes, respectively (fold change).

A total of 896 and 1, 311 DEGs were found in W_IAA_ vs. U_IAA_ and W_GA_ vs. U_GA_, respectively (Fig. S1a, b). KEGG pathway enrichment analysis showed that diterpenoid biosynthesis (ko00904) and phenylpropanoid biosynthesis (ko00940) were significantly enriched pathways in W_IAA_ vs. U_IAA_, but not in W_ut_ vs. U_ut_ and W_mock_ vs. U_mock_ (Fig. 5, Fig. S2). Most of IAA signal transduction genes (*ARG7s*, *IAA30*, *GH3.1*, and *SAUR71*), GA-related genes (*GA20OX3s, GA20OX1, GA30OX1s, GA20OX2, GA2OX8, AOP1, GAIs,* and *RGL*) and phenylpropanoid biosynthesis genes (*F5H*, *CCoAOMT*, *CCR*, *CADs*, and *POD*) were down-regulated in W_IAA_ (weeping stem on IAA treatment) compared to U_IAA_ (Fig. 6a). Those results above suggested different responses to IAA treatment between weeping and upright stems.

Genes involved in GA metabolism (*GA20OX3s*, *GA2OX8*, and *AOP1*) and GA signal transduction (*GAI*) gene, were both down-regulated in W_GA_ compared to U_GA_ (Fig. 6b, Table S7). In addition, pathways of ABC transporters (ko02010), glycosylphosphatidylinositol (GPI)-anchor biosynthesis (ko00563) were enriched in W_GA_ vs. U_GA_ rather than in W_ut_ vs. U_ut_ and W_mock_ vs. U_mock_ (Fig. 5a,b). Nine genes encoding ABC transporters, including four ABCB genes that were reported to participate in the hormone transport, having changed transcript levels in W_GA_ vs. U_GA_, while only three ABC transporter genes (*ABCB10, ABCB26, ABCF4*) changed in W_IAA_ vs. U_IAA_. Four genes related to GPI—anchor biosynthesis were up-regulated in W_GA_ (*PIGO*, *PIGT*, *PIGX*, and *PIGL*). Whole-wide genome predicted GPI-anchored proteins including proteins involved in cellulose metabolism (*EG*, *CSL*, *COBRA-like*), pectin metabolism (*UGDH, PG, PE, PL, PEM*), lignin biosynthesis (*laccase-7*), and ABC transporter (*ABCB4*) (Table S20). Moreover, the expression of four and two genes related to cellulose and pectin catabolism changed in W_IAA_ vs. U_IAA_, respectively (Fig. 6a); the expression of eight and five genes related to cellulose and pectin metabolism changed, respectively, in W_IAA_ vs. U_IAA_ (Fig. 6b).

### Validating gene expression patterns by qRT-PCR

To further validate the expression patterns of candidate genes, qRT-PCR was performed. Ten genes involved in phenylpropanoid metabolism (*Pm021214*), flavonoid metabolism (*Pm023086*), cell wall metabolism (*Pm027000*, *Pm025897*, *Pm019059*, *Pm015115*, *Pm023949*) as well as hormone metabolism and signal transduction (*Pm021879*, *Pm021015*, *Pm012245*) pathways, and two genes (*Pm024167*, *Pm024165*) located on the region of 10.56–11.68 Mb of chromosome 7, which were reported to be closely related to weeping traits, were selected to examine their expression levels in upright and weeping stems of *P. mume* (Fig. S4). The expression patterns of all 12 genes, *Pm021214* (*CAD*), *Pm023086* (*PLR*), *Pm027000* (*PE*), *Pm025897* (*PG*), *Pm019059* (*EXP*), *Pm015115* (*CSL*), *Pm023949* (*EG*), *Pm021879* (*AGR7*), *Pm021015* (*SAUR20*), *Pm012245* (*AOP1*), *Pm024167* (*SWEET4*), and *Pm024165* (*NPL6*), were in accordance with the trend of the expression data obtained by RNA-Seq.

## Discussion

**Figure 7:**
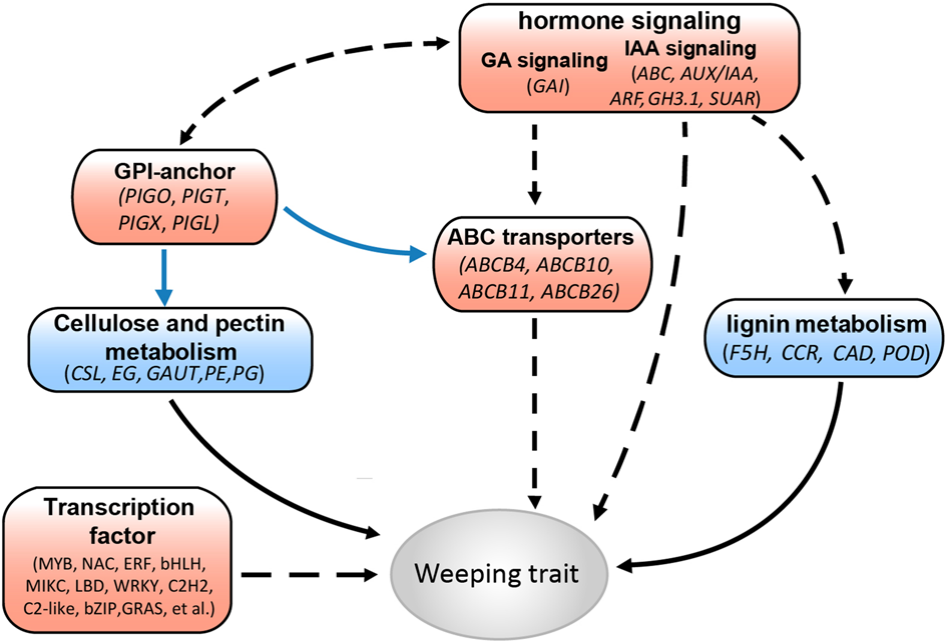
Summary of transcription-level regulation of the formation of the weeping trait in *P. mume.* The solid black line indicates direct control, the black dotted line indicates indirect regulation, and the solid blue line indicates post-transcriptional modification of proteins.

*Prunus mume* with weeping trait has highly ornamental and economic value because of its unique and weeping branch type. We found very different responses to IAA and GA_3_ between upright and weeping stems, and the weeping stem was deficient in phloem fibres and less developed in xylem compared with the upright stem. Moreover, the results of transcriptome analysis also suggested that several genes involved in cellulose, pectin and lignin biosynthesis, as well as multiple hormone metabolism and signal transduction pathway genes, were differentially expressed between the two stem types. Furthermore, after application of IAA, genes related to phenylpropanoid biosynthesis pathways have lower transcript levels in weeping stems, and most of IAA signal transduction genes, including *ARG7s*, *IAA30*, *GH3.1*, and *SAUR71*, have lower transcript levels in weeping stems than in upright stems. These results may be related with smaller angles of weeping stems responded to IAA treatment. After application of GA_3_, *GAI*, a gene of DELLA family that encodes a GA signal suppressor, has higher transcript levels in weeping stems than in upright stems, which is consistent with the results that weeping stems changed smaller angles than upright stems to respond to GA_3_ treatment. In addition, the transcript levels of phenylpropanoid biosynthesis, ABC transporters, and Glycosylphosphatidylinositol (GPI)—anchor biosynthesis genes vary in U_GA_ and W_GA_, and these genes may contribute to the differences in GA response between two stem types of *P. mume*. Thus, pendulous-stem traits may be due to the inability to respond to plant hormone signals normally and abnormal development of xylem and phloem fibres, thus resulting in reduced mechanical support and inability to keep growing upright. A hypothetical model for weeping trait formation in *P. mume* is summarized as Fig. 7.

Stem cross-sections displayed that although phloem portion of weeping stem increased, the xylem and phloem fibre portion of weeping stem was reduced compared with upright stem (Fig. 2). Instead of playing a mechanical support role, plant fibre with constitutively formed tertiary cell wall (G3 layer) inside the secondary cell wall was also reported to serve as ‘plant muscles’ and pull upward stem by fibre-cell shortening^24,25^. Mellerowicz et al. (2008) suggested that the structure of noncellulosic polysaccharides, such as hemicellulose and pectin, entrapped by laterally interacting cellulose microfibrils results in the tension to underpin the unique mechanical properties of fibres^25^. Xyloglucan, a kind of hemicellulose, was reported to be involved in restoring the vertical position of inclined poplar trees^25^. Although hemicellulose and cellulose contents are not significantly different in the abaxial side between weeping and upright stems, in the adaxial side, hemicellulose and cellulose contents in weeping stems were both higher than in upright stems^26^. The expression of several genes involved in cellulose (*Pm0150015*, *Pm023949*) and pectin (*Pm023569*, *Pm027000*, *Pm025897*) metabolism as well as other cell wall proteins (*Pm019059*, *Pm023337*) changed in weeping stems.

Lignin is another material that provides mechanical strength in the walls of sclerenchyma cells, such as tracheary elements^26^. In upright stems, the lignin content in the abaxial side is higher than that in adaxial side, which presented an opposite trend in weeping stem. In the adaxial side, the lignin content of weeping branches was higher than that in upright ones; in the abaxial side, the lignin content in weeping stems was lower than that in upright stems^26^. Transcriptome analysis also suggested that a number of genes related to lignin biosynthesis, such as *F5Hs*, *CCR*, *CADs*, *POD*, were down-regulated in weeping stems (W_ut_ vs. U_ut_), which may lead to lower lignin contents in weeping stems^27–30^. These results suggested that secondary growth changed, resulting in fewer xylem and phloem fibres in weeping stems. The decrease in xylem and phloem fibres in stems may reduce the mechanical support and affect the negative geotropic growth in weeping stems. *MYB*, *NAC*, *AP2/ERF*, *bHLH*, *LBD*, *WRKY*, *C2H2* were the transcription factor families with the largest number of DEGs between weeping and upright stems that might be involved in regulating weeping stem formation. The biosynthesis of lignin and cellulose is spatially and temporally regulated and is strongly associated with the sclerenchyma cell differentiation during plant growth and development. Previous studies have reported that several transcription factors, such as WRKY^31–33^, bHLH^34^, and C2H2-type zinc finger proteins^35^, MYB^36,37^, and NAC^38,39^, can regulate the temporal and spatial expression of secondary cell wall synthesis genes. There are 23 *MYB*, 15 *NAC*, and seven *WRKY*, nine *bHLH* and seven *C2H2* DEGs in W_ut_ vs. U_ut_, respectively.

Plant architecture is controlled by auxin and gibberellin^3,4,6,26^. Auxin plays key roles in regulating meristem development and secondary growth processes. A large number of genes associated with auxin and gibberellin show altered expression levels in *S. matsudana* with the weeping trait^3^. Distribution of GA in adaxial and abaxial shoots is uneven in weeping mulberry (*Morus alba* var. *pendula*) and *P. persica* var. *pendula* with the weeping trait^4, 6^. These findings have suggested that auxin and gibberellin are associated with weeping trait in multiple plants. Zhang et al. analyzed the hormone contents of upright and weeping stems in *P. mume* by LC–MS/MS^26^. In annual stems, IAA contents in weeping stems were higher than that in upright stems both in abaxial side and adaxial side. Although GA_3_ contents were not different between weeping and upright branches in base, the contents in the tip and middle of weeping branches were higher than that in upright branches. GA_3_ contents in the tip were higher than that in the middle and base of both weeping and upright branches^24^.

In *P. mume*, weeping stems had smaller and larger angles than upright stems in response to IAA and GA_3_ treatment, respectively. Additionally, transcriptome analysis also suggested that multiple hormone metabolism and signal transduction pathway genes were differentially expressed between two stem types. The differences in hormone content, hormone responses, and transcriptome between weeping and upright stems showed that IAA and GA participated in the formation of weeping trait in *P. mume*.

Auxin is a typical phytohormone involved in plant developmental processes such as embryo morphogenesis, cell division and elongation, vascular tissue differentiation, lateral root initiation, geotropism and phototropism, among others^40–46^. Previous studies have revealed that IAA, ARG, GH3.1, and SAURs are key proteins affecting gravitropic and auxin-mediated growth responses in *Arabidopsis*^42–44^. The asymmetric expression of *SAUR* genes in *Arabidopsis* facilitates gravitropism and phototropism of hypocotyls by promoting cell elongation^45, 46^. Several SAUR family genes, including *ARG7s* (*indole-3-acetic acid-induced protein*) (*Pm021879*, *Pm021884*, *Pm021062*, *Pm021877*, *Pm021896*) and *SAUR20* (*SMALL AUXIN UP RNA 20*, *Pm021015*), were down-regulated in weeping stems (W_ut_ vs. U_ut_) (Fig. 4). *GH3* genes, encoding IAA conjugating enzyme, participates in regulating auxin homeostasis. Overexpression of *GH3* genes reduced auxin levels and causes a dwarfed phenotype in *Arabidopsis*^47^. Two *GH3.1* genes (*Pm002438*, *Pm021243*) were up-regulated in weeping stems. Following IAA treatment, the transcript levels of six auxin-related genes in weeping stems were lower than that in upright stems (W_IAA_ vs U_IAA_), including *IAA30* (*Pm012868*), *ARGs* (*Pm021896*, *Pm021657*), *GH3.1* (*Pm002438*), and *SAURs* (*Pm021658*, *Pm013099*). *LAZY1* and *TAC1* were reported to regulate weeping traits by regulating polar auxin transport and light signal response in multiple species^12–17^, their expression levels were not significantly different between upright and weeping stems, but those genes still possibly contribute to the weeping trait via differential expression between adaxial and abaxial sides of the branch or their protein function is affected by gene mutation, such as single-nucleotide polymorphisms (SNPs) and insertion/deletion (indel) variants in weeping stems. For example, compared to standard peach growth habit, a variable simple sequence repeat (SSR) located within *TAC1* was disrupted and contributed to the protein structure changed in pillar peach trees^48^. In rice, an important mutation from AGGA to GGGA in the splicing site of the intron resulted in a *tac1* mutant with compact plant architecture and narrower tiller angle^49^.

Previous studies showed that auxin can promote the GA biosynthesis by maintaining the transcript level of *PsGA3ox1* in shoots of pea (*Pisum sativum*)^50^. After applying IAA instead of water on the stem, several GA biosynthesis genes were both up-regulated and diterpenoid biosynthesis pathways were enriched in W_IAA_ vs. W_mock_ and U_IAA_ vs. U_mock_, suggesting that IAA treatment may promote the GA synthesis in two kinds of stems (Table S12, Table S14, Fig. S2b, c). The diterpenoid biosynthesis genes were enriched and GA synthesis genes were down-regulated in W_IAA_ vs. U_IAA_ (Fig. 6a), indicating that IAA promotes GA synthesis in different degrees between weeping and upright stems.

Gibberellins (GAs) affect plant architecture by stimulating cell elongation and division in the stem^51^. GA metabolism gene *AOP1*, a homologous gene of *At1g52800* gene which encodes a 2-oxoglutarate-dependent dioxygenase AOP1 that is similar to gibberellin 20-oxidase^52^, was up-regulated in weeping stems. A putative gene encoding 2OG—Fe(II) oxygenase controlled the columnar—type growth in apple^53^. In addition, *GA2OX8*, a homologous gene of *AtGA2OX8* in *Arabidopsis*, was down-regulated in weeping stems. *AtGA2OX8* can negatively regulate the synthesis of bioactive GA via 2β-hydroxylated C20-GAs (GA_12_ and GA_53_) in *Arabidopsis*. Because 2β-hydroxylated C20-GA precursors can not be converted to activate GAs, overexpression of *AtGA2ox8* results in a decrease in active GA levels^54^. In weeping stems, GA biosynthesis genes were up-regulated and GA catabolism genes were down-regulated. Oddly, however, GA synthesis genes (*GA20OX3* and *GA3OX1)*, GA signal transduction gene *GAI*, and GA degradation genes, *GA2OX8* and *AOP1*, were down-regulated in both W_IAA_ vs. U_IAA_ and W_GA_ vs. U_GA_. These results may associate with the phenotypic hormone response that weeping stems were more sensitive to gibberellin treatment than upright stems. Previous studies showed that GA signals were associated with cell wall development in plants. GAI is a DELLA protein and a critical repressor of the GA response in *Arabidopsis* and the *gai-1* mutant, which exhibits excessive GA synthesis, resulting in a cytoskeletal defect and, thus, a reduction of cell length and thickness and cellulose and hemicellulose in the cell wall. Gibberellic acid can induce highly significant increases in cell diameter and wall thickness of problem fibres in *Triticum aestivum*^55^. In addition, after GA_3_ treatment, four genes involved in GPI-anchor biosynthesis had higher transcript levels in weeping stems than in upright stems. A large number of proteins related to lignin, cellulose, and pectin biosynthesis were found in predicted GPI-anchor proteins in *P. mume*, suggesting that GPI-anchored protein modification may connect with cell wall metabolism by regulating the activities of cellulose and pectin metabolism proteins. These features indicated that GA might participate in the biosynthesis of lignin and plant cell wall in *P. mume*, but the regulation mechanism still needs further study. A total of 11 genes are expressed differentially in three comparisons and ABC transporter pathway was significantly enriched in W_GA_ vs. U_GA_. *ABCB1*, *ABCB4*, *ABCB10*, *ABCB11*, *ABCB14*, *ABCB15*, *ABCB19*, and *ABCB21,* members of ABCB subfamily, have been well characterized as auxin transporters and several ABCB genes are involved in stem development in *Arabidopsis*^56–58^. *XLOC_004438*, *Pm008507*, and *Pm008652* were homologous genes of *ABCB10*, *ABCB11*, and *ABCB4* in *Arabidopsis*, respectively, which were both down-regulated in W_GA_ vs. U_GA_. In *Arabidopsis*, *AtABCG14/36/38*, belonging to ABCG subfamily, also joined in the transport of hormones and growth-regulating substances. *AtABCG14* could deliver cytokinin from roots to shoots^59^, while *AtABCG36* took part in regulating the intracellular accumulation of indole-3-butyric acid (IBA), the storage precursor of indole-3-acetic acid (IAA), by mediating its efflux^60^. Moreover, several ABCG transporters also regulated vascular development. *AtABCG29* participated in the lignin monomer transport process^61^, and *ABCG9/11/14* were essential to vascular development^62^. In *P. mume*, the expression level of *Pm004997*, a homology gene of *AtABCG39*, was decreased 2.8- and 5- fold in W_ut_ vs. U_ut_ and W_GA_ vs. U_GA_, respectively (Table S5, Table S7, Fig. 6b).

Our recent studies revealed that weeping trait might be controlled by a major gene and multiple minor genes based on the character separation ratio of F_1_ generation^26^. In order to investigate the major gene that controlled the weeping trait, several analyses were conducted. QTL analysis of F_1_ generation showed that weeping trait was associated with the genes in 7.80–87.65 cM of chromosome 7, nearly covered chromosome 7. In order to find the exact location of the major locus, Mutmap strategy and calculation of the recombination rate between the weeping trait marker (marker 0) and other SLAF markers were conducted. The results showed that the major gene that controlled weeping trait might be located on the region of 10.56–11.68 Mb of chromosome 7. A total of 28 DEGs, including *PEM* (*pectin methylesterase*, *Pm023569*), *EXLB1* (*Pm023337*), *EG* (*endoglucanase*, *Pm023949*), between upright and weeping stems on the chromosome 7 were extracted and listed in Table S21. Importantly, three DEGs (*Pm024165*, *Pm024167*, *Pm024338*) located on the region of 10.56–11.68 Mb of chromosome 7 and might be candidate major genes. *Pm024165* (NLP6, NIN-LIKE PROTEIN 6) is a transcription factor and the homology with *AT1G64530* genes that regulate Nitrate signal in *Arabidopsis*^63^; *Pm024338* encodes a C2 and GRAM domain-containing protein which is homologous with *AT5G50170* in *Arabidopsis*, a function unknown protein. *Pm024167* is a homology gene of *Arabidopsis SWEET4* which located on the plasma membrane and served to transport glucose from source organs to sink tissues through the phloem translocation pathway. The down-regulated expression and knock-down of *SWEET4* in *Arabidopsis* leaded to the defects in glucose and fructose transporter and reduction in glucose and fructose contents^64^. Glucose is a raw material of polysaccharide synthesis, and its decreased transcript levels may influence the synthesis of hemicellulose, cellulose, and pectin, leading to the weeping traits in *P. mume*. *Pm024165*, *Pm024167*, *Pm024338* may be the candidate genes that lead to the formation of weeping stems in *P. mume*, but whether one of the three genes is the weeping trait major gene still need further study, because some factors, such as protein structure and protein post-translational modification, also affect protein function and plant phenotype. On the other hand, owing to phytohormones, cell wall, and phenylpropanoid metabolism pathways may be influenced in weeping stems, so DEGs involved in those pathways in W_ut_ vs. U_ut_ may work as candidate minor genes to contribute to the weeping trait.

## Methods

### Plant materials and treatments

One month after bud germination in spring, elongating juvenile stems shorter than 10 cm from seven upright and weeping grafting progenies were selected from five year old F_1_ population of *P. mume* 'Liuban' × 'Fentai Chuizhi' in greenhouse of Beijing Forestry University, respectively. Lanolin containing water, 2 mg/L GA_3_ and IAA were applied to the adaxial side of the stems in the elongation zone 2 cm from the stem tip. The delayed photography of 400 min after treatment were taken by Canon EOS 80D camera (Canon, Japan), and Image J software (National Institute of Health, USA) was used to compare the photos of 0 and 360 min (6 h) and to calculate their deflection angles (Fig. S5). After 6 h of water, IAA or GA_3_ treatment, seven stem tips 1 cm in length from upright and weeping progenies were collected and mixed for RNA-seq with three biological repeats. All samples were immediately frozen in liquid nitrogen and stored at -80 °C for further usage.

### Histochemical and histological analyses

To observe the anatomical differences of the lignified stem between weeping and upright progenies, 0.5-cm stems in the middle of the elongating annual upright and weeping stems of *P. mume* were fixed in formaldehyde-acetic acid solution [formaldehyde:glacial acetic acid: 70% ethanol (1:1:18)] for 24 h, dehydrated in a graded ethanol series, and embedded in paraplast. The samples were sectioned at a thickness of 8 μm using a Leica RM2235 rotary microtome. The sections were stained with safranin and fast green and then screened using a Pannoramic SCAN scanner (3DHISTECH, Budapest, Hungary). The free-hand section from lignified annual stem with upright and weeping traits, respectively, was stained with hydrochloric acid—phloroglucinol solution and then observed and photographed under a stereoscopic microscope (Leica EZ4 HD) (Leica, Germany). Stem cross section, xylem, phloem, and pith areas were measured using Image J software, and the calculation formulas of different tissue proportions are as follows:

1 $${\text{Xylem area proportion}} = \left( {\text{Xylem area}} \right)/\left( {\text{Stem cross sectional area}} \right)$$

2 $${\text{Phloem area proportion}} = \left( {\text{Phloem area}} \right)/\left( {\text{Stem cross sectional area}} \right)$$

3 $${\text{Pith area proportion}} = \left( {\text{Pith area}} \right)/\left( {\text{Stem cross sectional area}} \right)$$

4 $${\text{Phloem fibre area proportion}} = \left( {\text{Phloem fibre area}} \right)/\left( {\text{Stem cross sectional area}} \right)$$

### RNA extraction, library construction, RNA-seq and genome alignment

Total RNA of all stem samples was extracted with the Plant Total RNA Kit (Omega Bio-Tek, Norcross, GA, USA). RNA concentration and quality were determined using a NanoDrop ND1000 (Thermo Scientific, USA) and electrophoresis on formaldehyde-containing 1% agarose gels. Approximately 3 μg of total RNA from each sample (U_ut_, W_ut_, U_IAA_, W_IAA_, U_GA_, and W_GA_) was enriched by Oligo (dT) beads and broken into short fragments for library construction according to operating instructions. Then the cDNA library was used for sequencing by Illumina HiSeq 2500 (Illumina, Santiago, California, USA). The obtained clean sequencing data were aligned with the *P. mume* genome using TopHat2 (http://ccb.jhu.edu/software/tophat/index.shtml)^65^.

All assembled unigenes were BLASTed in KEGG ortholog database (KO) and Gene onthology (GO) databases using BLAST2GO with a cut-off E-value of 10^–6^ ^66–69^. Differentially expressed genes (DEG) were identified when the FDR (false discovery rates) < 0.05 and absolute value of |log2 Fold Change|≥ 1. Furthermore, DEGs were also annotated to perform functional category analysis using the MapMan Mercator tool (http://mapman.gabipd.org /web/guest/mercator).

### Validation of RNA-seq data by qRT-PCR

The transcript levels of 12 genes in six tissues (U_ut_, W_ut_) were examined using qRT-PCR. Total RNA was extracted using the RNA extraction kit (Tiangen, Beijing, China) following the manufacturer’s instructions to synthesize first-strand cDNA using the PrimerScript RT Reagent Kit (TaKaRa, Dalian, China). Gene-specific primers were designed by IDT (https://sg.idtdna.com/scitools/Applications/RealTimePCR/) based on the gene sequences from the *P. mume* genome, which are listed in Table S22. The fluorescent dye SYBR Green II (TaKaRa) was applied in the detection system, and *PmPP2A* was selected as a reference gene according to previous reports^70,71^. A 7500 Real-Time PCR System (Applied Biosystems, USA) was used to conduct a three-step PCR procedure. Three biological replicates were carried out, and transcript levels were calculated by the 2^−ΔΔCt^ method^72^.

### GPI—anchored protein prediction

Proteins containing ω—site were predicted among whole-wide genome of *P. mume* using software PredGPI (http://gpcr.biocomp.unibo.it/predgpi/pred.htm) with specificity ≥ 99.5^73^.

### Statistical analysis

All data in the text were tested by analysis of variance (ANOVA) using SPSS version 11.0. Least significant differences (LSDs) were calculated to compare significant effects at the 5% level.

## Conclusions

The morphological and histochemical characteristics of the upright and weeping stems of *P. mume* revealed defects in the xylem and phloem fibres in weeping stems. Compared to upright stems, weeping stems were more sensitive to GA_3_ and less sensitive to IAA. Furthermore, comparative analysis of transcriptome data revealed that phenylpropanoid biosynthesis, cellulose and pectin biosynthesis, and phytohormone signal transduction pathways were altered in two stem types. Most of IAA signal transduction genes, including *ARF7s*, *IAA30*, *GH 3.1*, and *SAUR71*, and GA metabolism genes, have lower transcript levels in weeping stems than in upright stems. After application of GA_3_, genes involved in phenylpropanoid biosynthesis, ABC transporters, and Glycosylphosphatidylinositol (GPI)—anchor biosynthesis genes were differentially expressed between upright and weeping stems. Our study provides a theoretical reference for the molecular mechanism analysis of weeping trait in *P. mume*.

## Supplementary information

Supplementary Information 1

Supplementary Information 2

Supplementary Information 3

Supplementary Information 4

Supplementary Information 5

Supplementary Information 6

Supplementary Information 7

Supplementary Information 8

Supplementary Information 9

Supplementary Information 10

Supplementary Information 11

Supplementary Information 12

Supplementary Information 13
