# Supplementary material for: Comparative gene expression analysis reveals that multiple mechanisms regulate the weeping trait in Prunus mume
Source: Sci Rep. 2021 Jan 29;11:2675. doi: 10.1038/s41598-021-81892-3 (PMC7846751; doi:10.1038/s41598-021-81892-3)
Supplement: Supplementary file 1 — Supplementary Information 1 [file 41598_2021_81892_MOESM1_ESM.docx]

**Comparative gene expression analysis reveals that multiple mechanisms regulate together the weeping trait in *Prunus mume***

**Lulu Li^+^, Yichi Zhang^+^, Tangchun Zheng*, Xiaokang Zhuo, Ping Li, Like Qiu, Weichao Liu, Jia Wang, Tangren Cheng and Qixiang Zhang***

Beijing Advanced Innovation Center for Tree Breeding by Molecular Design, Beijing Key Laboratory of Ornamental Plants Germplasm Innovation & Molecular Breeding, National Engineering Research Center for Floriculture, Beijing Laboratory of Urban and Rural Ecological Environment, Engineering Research Center of Landscape Environment of Ministry of Education, Key Laboratory of Genetics and Breeding in Forest Trees and Ornamental Plants of Ministry of Education, School of Landscape Architecture, Beijing Forestry University, Beijing, 100083, China

*Correspondence: zhengtangchun@bjfu.edu.cn; zqxbjfu@126.com

^+^these authors contributed equally to this work

**1 SUPPLEMENTARY DATA**

Supplementary Figure S1. Analysis of differentially expressed genes (DEGs) in branches between upright and weeping branches following the different treatments.

Supplementary Figure S2. KEGG enrichment analysis of DEGs in W_mock_ vs. U_mock_, U_IAA_ vs. U_mock_, W_IAA_ vs. W_mock_, U_GA_ vs. U_mock_, W_GA_ vs. W_mock_.

Supplementary Figure S3. MapMan Bins of “Metabolism_overview” in three comparisons.

Supplementary Figure S4. qRT-PCR validation of 12 DEGs in W_ut_, U_ut_, W_IAA_, U_IAA_, W_GA_, and U_GA_.

Supplementary Figure S5. Diagram of deviation angle measurement after 400 min treatment.

Supplementary Table S1. Differences between upright and weeping branches in response to hormone treatment with different concentrations.

Supplementary Table S2. Statistics of RNA-seq reads and genome alignment in 24 samples.

Supplementary Table S3. Statistics of gene number in 24 samples.

Supplementary Table S4. The total information of DEGs in W_mock_ vs. U_mock_.

Supplementary Table S5. The total information of DEGs in W_ut_ vs. U_ut_.

Supplementary Table S6. The total information of DEGs in W_IAA_ vs. U_IAA_.

Supplementary Table S7. The total information of DEGs in W_GA_ vs. U_GA_.

Supplementary Table S8. All DEGs in three comparisons in profiles 0-19.

Supplementary Table S9. Significantly enriched GO pathways of DEGs in W_ut_ vs. U_ut_.

Supplementary Table S10. KEGG annotation of DEGs in W_ut_ vs. U_ut_.

Supplementary Table S11. DETFs in W_ut_ vs. U_ut_.

Supplementary Table S12. The total information of DEGs in W_IAA_ vs. W_mock_.

Supplementary Table S13. KEGG annotation of DEGs in W_IAA_ vs. W_mock_.

Supplementary Table S14. The total information of DEGs in U_IAA_ vs. U_mock_.

Supplementary Table S15. KEGG annotation of DEGs in U_IAA_ vs. U_mock_.

Supplementary Table S16. The total information of DEGs in W_GA_ vs. W_mock_.

Supplementary Table S17. KEGG annotation of DEGs in W_GA_ vs. W_mock_.

Supplementary Table S18. The total information of DEGs in U_GA_ vs. U_mock_.

Supplementary Table S19. KEGG annotation of DEGs in U_GA_ vs. U_mock_.

Supplementary Table S20. Genome-wide GPI-anchored protein prediction in *P. mume*.

Supplementary Table S21. DEGs between upright and weeping branches and located on the chromosome 7

Supplementary Table S22. Primer sequences used for qRT-PCR.

**2 SUPPLEMENTARY FIGURES**


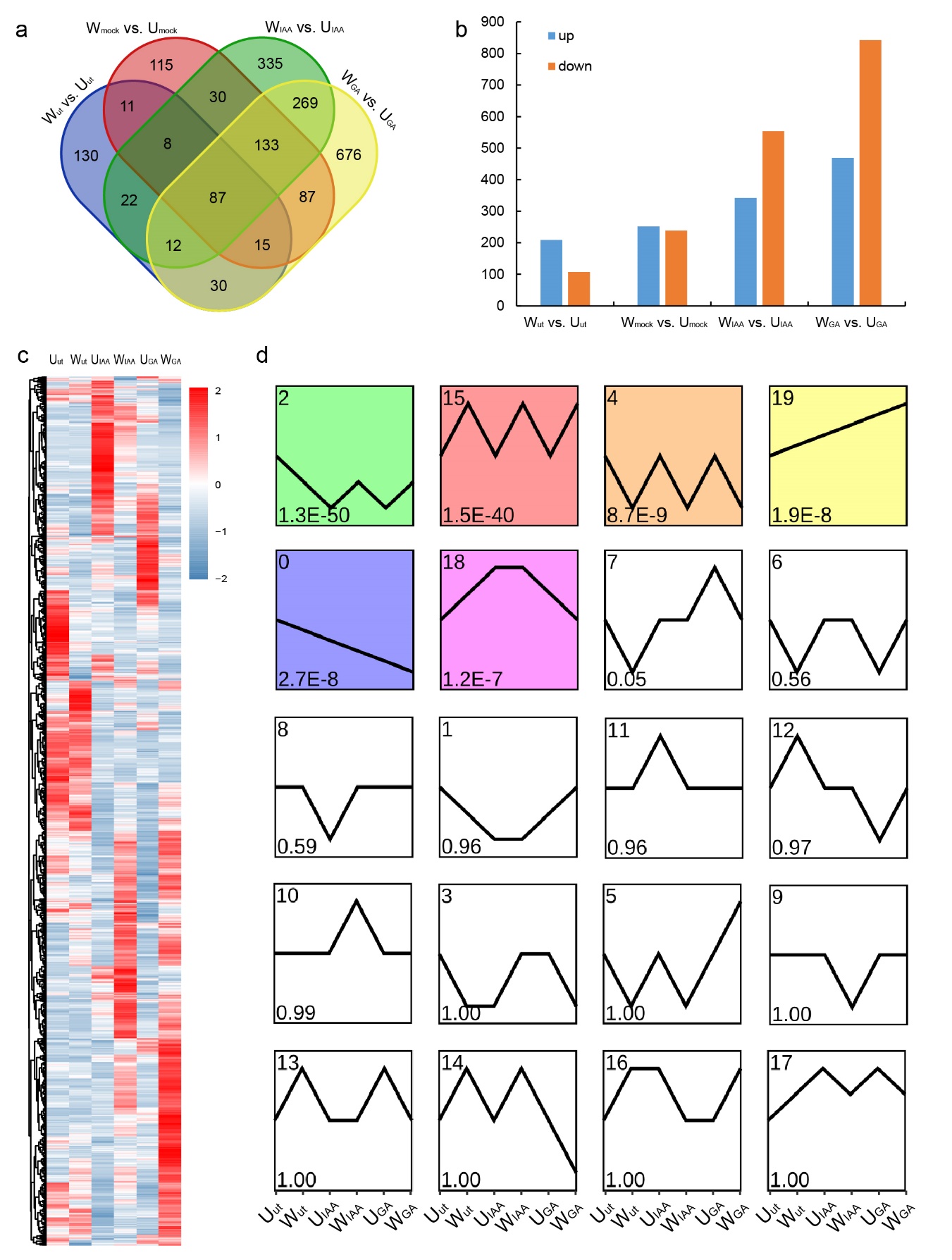


**Figure S1.** Analysis of differentially expressed genes (DEGs) in branches between upright and weeping branches following the different treatments. (**a**) Venn diagram of the number of DEGs (*P* value < 0.05) between the four comparison (W_ut_ vs. U_ut_, W_mock_ vs. U_mock_, W_IAA_ vs. U_IAA,_ W_GA_ vs. U_GA_). (**b**) Number of up-regulated and down-regulated DEGs between the four comparisons (W_ut_ vs. U_ut_, W_mock_ vs. U_mock_, W_IAA_ vs. U_IAA,_ W_GA_ vs. U_GA_). (**c**) Overall cluster of DEGs of six libraries (W_ut_, U_ut_, W_IAA_, U_IAA_, W_GA_, U_GA_). FPKM (fragments per kilobase of transcript per million base pairs sequenced) was used to estimate the level of gene expression. The colour change from red (highly expressed) to blue (low expressed) represents the log2 relative expression level values (FPKM). (**d**) Expression trend analysis of DEGs in three comparisons (W_ut_, U_ut_, W_IAA_, U_IAA_, W_GA_, U_GA_), profiles 0-19. The mean transcript levels of all genes in each of the six categories are displayed relative to log2 (FPKM). Numbers of DEGs and p-values in each profile are displayed, and the profiles with colour represent the trends of significant enrichment. U_ut_, untreated upright branches; W_ut_, untreated weeping branches; U_mock_, upright branches with water treatment; W_mock_, weeping branches with water treatment; U_IAA_, upright branches with IAA treatment; W_IAA_, weeping branches with IAA treatment; U_GA_, upright branches with GA_3_ treatment; W_GA_, weeping branches with GA_3_ treatment.


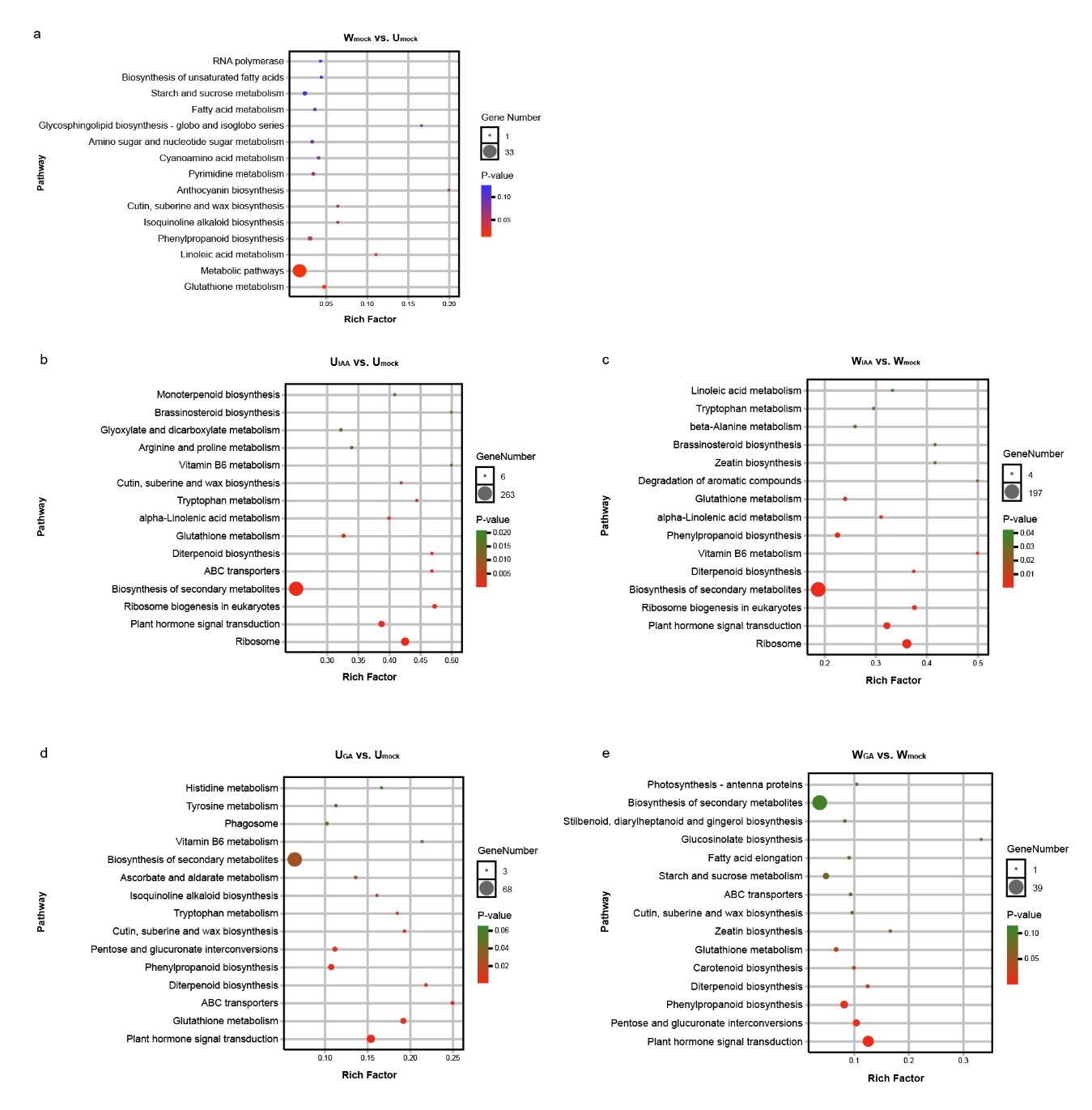


**Figure S2.** KEGG enrichment analysis of DEGs in W_mock_ vs. U_mock_, U_IAA_ vs. U_mock_, W_IAA_ vs. W_mock_, U_GA_ vs. U_mock_, W_GA_ vs. W_mock_. a, unique DEGs in W_mock_ vs. U_mock_; b, DEGs in U_IAA_ vs. U_mock_; c, DEGs in W_IAA_ vs. W_mock_; d, DEGs in U_GA_ vs. U_mock_; e, DEGs in W_GA_ vs. W_mock_.


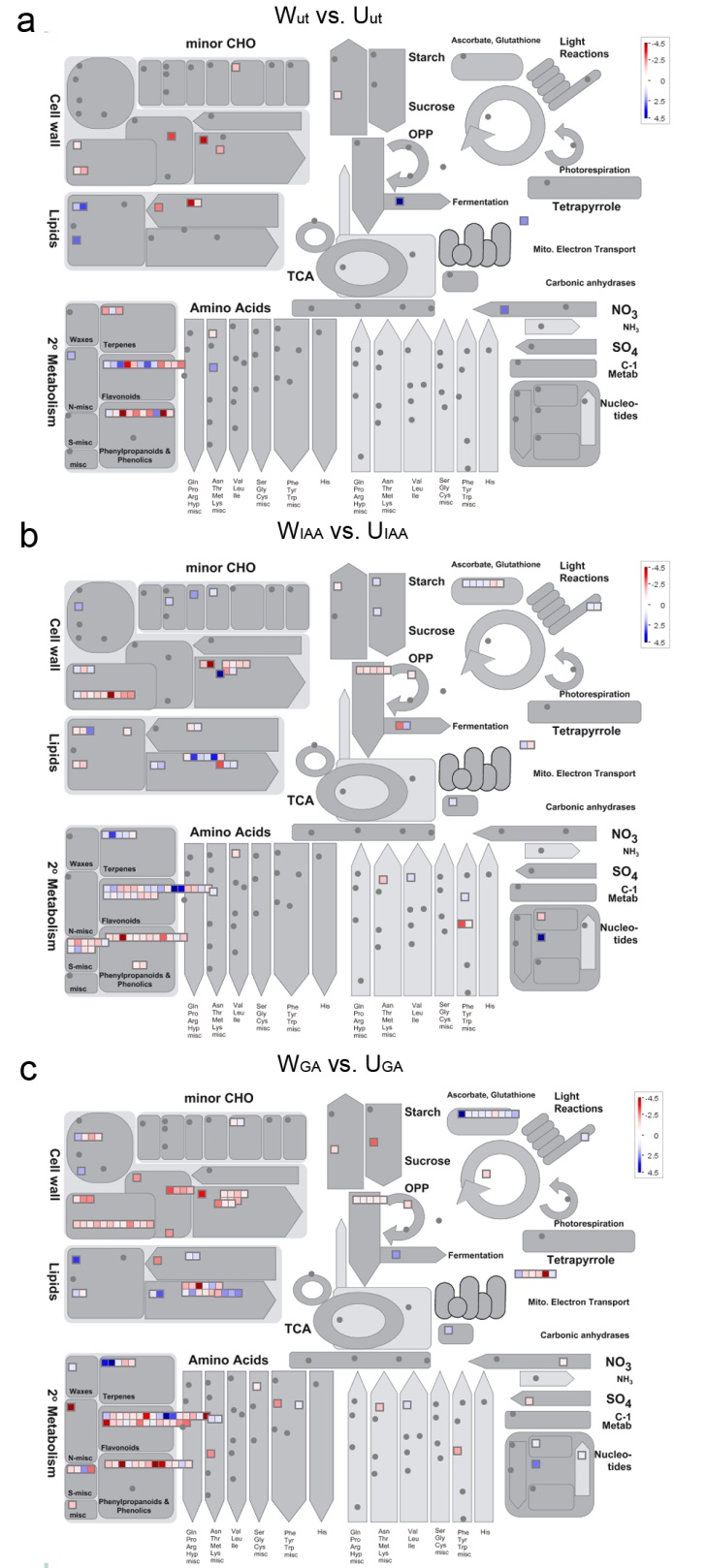


**Figure S3.** MapMan Bins of “Metabolism_overview” in three comparisons. (**a**) W_ut_ vs. U_ut_, (b) W_IAA_ vs. U_IAA,_ (c) W_GA_ vs. U_GA_.


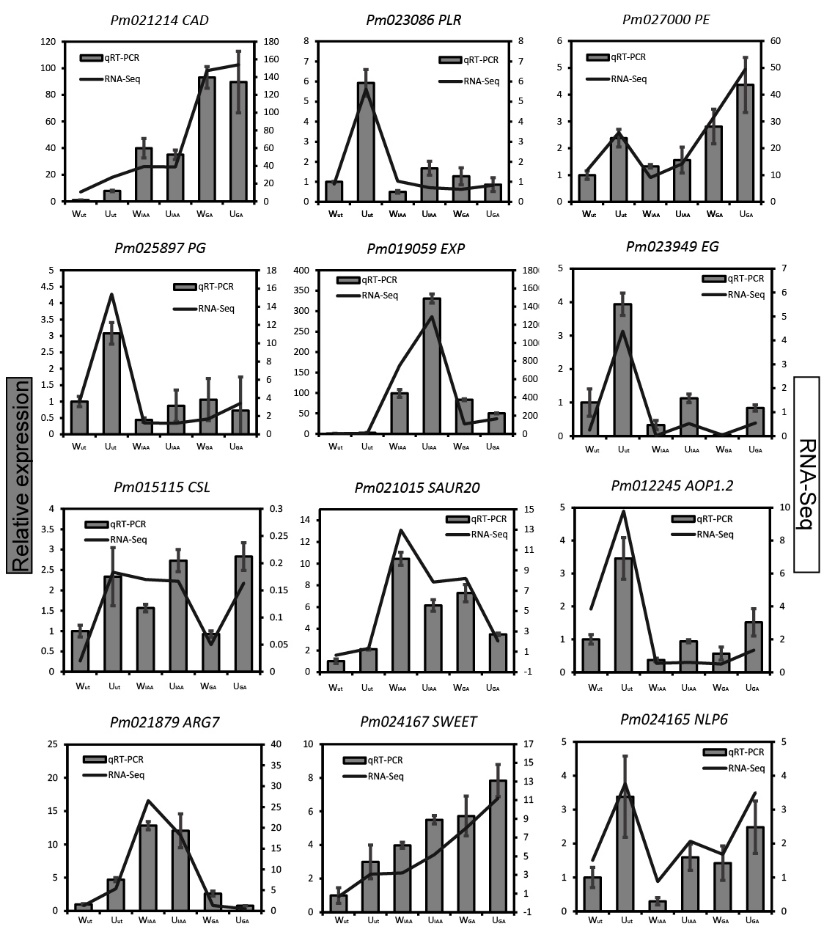


**Figure S4.** qRT-PCR validation of 12 DEGs in W_ut_, U_ut_, W_IAA_, U_IAA_, W_GA_, and U_GA_. Y-axes in the graphs show normalized fold changes in qRT-PCR (left) and FPKM in RNA-seq (right). qRT-PCR transcript levels were normalized to *PP2A*. Different letters indicate a significant difference (*P* < 0.05) based on a one-way ANOVA. Error bars represent one standard error of the mean (n = 3).


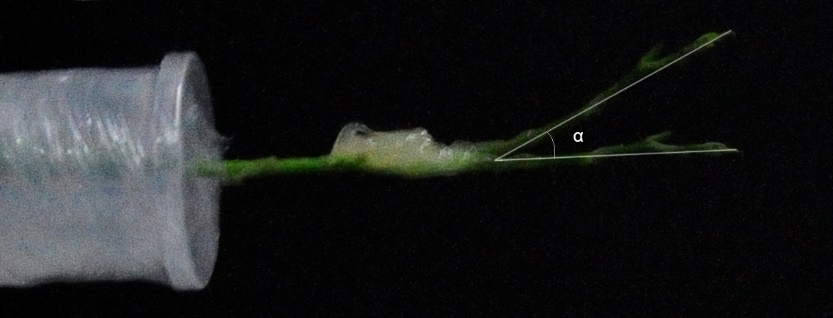


**Figure S5.** Diagram of deviation angle measurement after 400 min treatment.

**3 SUPPLEMENTARY TABLE**

Table S1. Differences between upright and weeping branches in response to hormone treatment with different concentrations.

| Treatment | Weeping branches | Upright branches |
| --- | --- | --- |
| IAA 1mg/L | (+) 1.83±0.20^a^ | (+) 3.37±0.13^a^ |
| GA_3_ 1mg/L | (+) 3.35±0.98^a^ | (+) 3.45±0.51^a^ |
| IAA 2mg/L | (+) 1.65 ± 0.17^b^ | (+) 10.73 ± 0.25^a^ |
| GA_3_ 2mg/L | (+) 13.99 ± 0.31^a^ | (+) 9.00 ± 0.29^b^ |
| IAA 3mg/L | (+) 41.19±2.66^b^ | (+) 86.44±1.76^a^ |
| GA_3_ 3mg/L | (+) 27.08±4.68^a^ | (+) 23.93±3.19^a^ |

Supplementary Table S2. Statistics of RNA-seq reads and genome alignment in 24 samples.

| Samples | Clean reads (bp) | Clean reads number | GC Content (%) | ≥ Q30 (%) | Mapped tags (%) |
| --- | --- | --- | --- | --- | --- |
| CKU-1 | 8314541400 | 55430276 | 46.44 | 94.90 | 80.57 |
| CKU-2 | 7629604200 | 50864028 | 46.52 | 94.97 | 80.44 |
| CKU-3 | 8923007100 | 59486714 | 46.50 | 95.09 | 80.35 |
| CKW-1 | 8257029000 | 55046860 | 47.54 | 94.25 | 81.13 |
| CKW-2 | 7516909200 | 50112728 | 46.48 | 94.71 | 80.78 |
| CKW-3 | 8486304900 | 56575366 | 46.48 | 94.83 | 80.48 |
| TU1-1 | 9110027700 | 60733518 | 46.68 | 96.20 | 79.74 |
| TU1-2 | 7572962400 | 50486416 | 46.20 | 96.21 | 79.61 |
| TU1-3 | 8278043400 | 55186956 | 46.63 | 96.16 | 79.60 |
| TW1-1 | 8048803200 | 53658688 | 46.15 | 96.33 | 80.07 |
| TW1-2 | 8509310400 | 56728736 | 46.31 | 96.18 | 79.12 |
| TW1-3 | 8051092500 | 53673950 | 46.23 | 96.38 | 79.73 |
| TU2-1 | 8119683600 | 54131224 | 46.75 | 94.55 | 80.77 |
| TU2-2 | 7457310600 | 49715404 | 47.09 | 94.49 | 80.61 |
| TU2-3 | 7071801300 | 47145342 | 47.19 | 94.54 | 80.85 |
| TW2-1 | 8471283900 | 56475226 | 46.52 | 94.93 | 80.83 |
| TW2-2 | 9118583100 | 60790554 | 46.42 | 95.00 | 81.16 |
| TW2-3 | 8104435800 | 54029572 | 46.24 | 95.06 | 80.72 |
| TU3-1 | 7203966900 | 48026446 | 46.99 | 94.13 | 79.83 |
| TU3-2 | 6694846200 | 44632308 | 47.43 | 94.27 | 79.52 |
| TU3-3 | 7610404200 | 50736028 | 47.31 | 94.54 | 80.03 |
| TW3-1 | 8010645600 | 53404304 | 46.56 | 94.92 | 79.45 |
| TW3-2 | 7442777400 | 49618516 | 46.52 | 94.84 | 79.44 |
| TW3-3 | 8332280100 | 55548534 | 46.39 | 94.92 | 79.89 |

Supplementary Table S3. Statistics of gene number in 24 samples.

| Sample name | Known Gene Number | New Gene Number |
| --- | --- | --- |
| CKU-1 | 17958 (94.00%) | 1147 (6.00%) |
| CKU-2 | 17902 (93.96%) | 1151 (6.04%) |
| CKU-3 | 17966 (93.91%) | 1166 (6.09%) |
| CKW-1 | 17852 (94.20%) | 1100 (5.80%) |
| CKW-2 | 17859 (94.06%) | 1128 (5.94%) |
| CKW-3 | 17876 (93.96%) | 1149 (6.04%) |
| TU1-1 | 18004 (93.90%) | 1171 (6.10%) |
| TU1-2 | 17920 (93.87%) | 1171 (6.13%) |
| TU1-3 | 17957 (93.90%) | 1167 (6.10%) |
| TW1-1 | 17912 (93.92%) | 1160 (6.08%) |
| TW1-2 | 17922 (93.85%) | 1175 (6.15%) |
| TW1-3 | 17941 (93.84%) | 1177 (6.16%) |
| TU2-1 | 17905 (93.91%) | 1162 (6.09%) |
| TU2-2 | 17894 (93.96%) | 1150 (6.04%) |
| TU2-3 | 17812 (94.00%) | 1138 (6.00%) |
| TW2-1 | 17916 (93.86%) | 1173 (6.14%) |
| TW2-2 | 18000 (93.87%) | 1176 (6.13%) |
| TW2-3 | 17870 (93.88%) | 1165 (6.12%) |
| TU3-1 | 17845 (94.12%) | 1134 (5.98%) |
| TU3-2 | 17788 (94.08%) | 1120 (5.92%) |
| TU3-3 | 17836 (94.01%) | 1137 (5.99%) |
| TW3-1 | 17920 (93.81%) | 1182 (6.19%) |
| TW3-2 | 17835 (93.85%) | 1168 (6.15%) |
| TW3-3 | 17986 (93.87%) | 1174 (6.13%) |

Supplementary Table S4. The total information of DEGs in W_mock_ vs. U_mock_.

In the individual excel format “Supplementary Table S4”

Supplementary Table S5. The total information of DEGs in W_ut_ vs. U_ut_.

In the individual excel format “Supplementary Table S5”

Supplementary Table S6. The total information of DEGs in W_IAA_ vs. U_IAA_.

In the individual excel format “Supplementary Table S6”

Supplementary Table S7. The total information of DEGs in W_GA_ vs. U_GA_.

In the individual excel format “Supplementary Table S7”

Supplementary Table S8. All DEGs in three comparisons in profiles 0-19.

In the individual excel format “Supplementary Table S8”

Supplementary Table S9. Significantly enriched GO pathways of DEGs in W_ut_ vs. U_ut_.

| Category | GO ID | Description | Gene Ratio (31) | BgRatio (2557) | P value | P.adjust |
| --- | --- | --- | --- | --- | --- | --- |
| biological processes | GO:0006468 | protein phosphorylation | 4 | 70 | 0.009275 | 0.881162378 |
| biological processes | GO:0016310 | phosphorylation | 4 | 90 | 0.021829 | 0.99921197 |
| molecular functions | GO:0016798 | hydrolase activity, acting on glycosyl bonds | 4 | 77 | 0.009326 | 0.235772358 |
| molecular functions | GO:0004312 | fatty acid synthase activity | 1 | 1 | 0.011052 | 0.235772358 |
| molecular functions | GO:0016297 | acyl-[acyl-carrier-protein] hydrolase activity | 1 | 1 | 0.011052 | 0.235772358 |
| molecular functions | GO:0004553 | hydrolase activity, hydrolyzing O-glycosyl compounds | 3 | 64 | 0.032195 | 0.397753674 |
| molecular functions | GO:0016790 | thiolester hydrolase activity | 1 | 3 | 0.032803 | 0.397753674 |
| molecular functions | GO:0004558 | alpha-1,4-glucosidase activity | 1 | 4 | 0.043504 | 0.397753674 |
| molecular functions | GO:0090599 | alpha-glucosidase activity | 1 | 4 | 0.043504 | 0.397753674 |

Supplementary Table S10. KEGG annotation of DEGs in W_ut_ vs. U_ut_.

| Pathway | W_ut_-vs-U_ut_ | All | P value | Q value | Pathway ID |
| --- | --- | --- | --- | --- | --- |
| Plant hormone signal transduction | 9 | 214 | 0.006075 | 0.407 | ko04075 |
| Biosynthesis of secondary metabolites | 25 | 1049 | 0.015381 | 0.515258 | ko01110 |
| Phenylpropanoid biosynthesis | 7 | 195 | 0.033193 | 0.636223 | ko00940 |
| Alanine, aspartate and glutamate metabolism | 3 | 48 | 0.039481 | 0.636223 | ko00250 |

Supplementary Table S11. DETFs in W_ut_ vs. U_ut_.

In the individual excel format “Table Supplementary Table S11”

Supplementary Table S12. The total information of DEGs in W_IAA_ vs. W_mock_.

In the individual excel format “Supplementary Table Table S12”

Supplementary Table S13. KEGG annotation of DEGs in W_IAA_ vs. W_mock_.

| Pathway | W_mock_-vs-W_IAA_ | All | Pvalue | Qvalue | Pathway ID |
| --- | --- | --- | --- | --- | --- |
| Ribosome | 106 | 296 | 9.11E-21 | 1.02E-18 | ko03010 |
| Plant hormone signal transduction | 69 | 214 | 6.85E-11 | 3.84E-09 | ko04075 |
| Ribosome biogenesis in eukaryotes | 35 | 93 | 5.79E-08 | 2.16E-06 | ko03008 |
| Biosynthesis of secondary metabolites | 197 | 1049 | 9.54E-05 | 2.67E-03 | ko01110 |
| Diterpenoid biosynthesis | 12 | 32 | 0.001523 | 3.41E-02 | ko00904 |
| Vitamin B6 metabolism | 7 | 14 | 0.002217 | 4.14E-02 | ko00750 |
| Phenylpropanoid biosynthesis | 44 | 195 | 0.002853 | 4.57E-02 | ko00940 |
| alpha-Linolenic acid metabolism | 14 | 45 | 0.004803 | 6.72E-02 | ko00592 |
| Glutathione metabolism | 25 | 104 | 0.009865 | 1.23E-01 | ko00480 |
| Degradation of aromatic compounds | 4 | 8 | 0.02152 | 2.25E-01 | ko01220 |
| Brassinosteroid biosynthesis | 5 | 12 | 0.024112 | 2.25E-01 | ko00905 |
| Zeatin biosynthesis | 5 | 12 | 0.024112 | 2.25E-01 | ko00908 |
| beta-Alanine metabolism | 13 | 50 | 0.030115 | 2.59E-01 | ko00410 |
| Tryptophan metabolism | 8 | 27 | 0.040058 | 3.15E-01 | ko00380 |
| Linoleic acid metabolism | 5 | 18 | 0.042239 | 3.15E-01 | ko00591 |

Supplementary Table S14. The total information of DEGs in U_IAA_ vs. U_mock_.

In the individual excel format “Supplementary Table S14”

Supplementary Table S15. KEGG annotation of DEGs in U_IAA_ vs. U_mock_.

| Pathway | U_mock_-vs-U_IAA_ | All | Pvalue | Qvalue | Pathway ID |
| --- | --- | --- | --- | --- | --- |
| Ribosome | 126 | 296 | 6.06E-20 | 7.09E-18 | ko03010 |
| Plant hormone signal transduction | 83 | 214 | 8.96E-11 | 5.24E-09 | ko04075 |
| Ribosome biogenesis in eukaryotes | 44 | 93 | 2.43E-09 | 9.48E-08 | ko03008 |
| Biosynthesis of secondary metabolites | 263 | 1049 | 4.65E-06 | 1.36E-04 | ko01110 |
| Diterpenoid biosynthesis | 15 | 32 | 0.000576 | 1.12E-02 | ko00904 |
| ABC transporters | 15 | 32 | 0.000576 | 1.12E-02 | ko02010 |
| Glutathione metabolism | 34 | 104 | 0.001583 | 2.47E-02 | ko00480 |
| alpha-Linolenic acid metabolism | 18 | 45 | 0.001692 | 2.47E-02 | ko00592 |
| Tryptophan metabolism | 12 | 27 | 0.003562 | 4.63E-02 | ko00380 |
| Cutin, suberine and wax biosynthesis | 13 | 31 | 0.004521 | 5.29E-02 | ko00073 |
| Vitamin B6 metabolism | 7 | 14 | 0.011922 | 1.27E-01 | ko00750 |
| Arginine and proline metabolism | 17 | 50 | 0.014897 | 1.45E-01 | ko00330 |
| Glyoxylate and dicarboxylate metabolism | 20 | 62 | 0.01608 | 1.45E-01 | ko00630 |
| Brassinosteroid biosynthesis | 6 | 12 | 0.019886 | 1.62E-01 | ko00905 |
| Monoterpenoid biosynthesis | 8 | 22 | 0.020724 | 1.62E-01 | ko00902 |
| Phenylpropanoid biosynthesis | 50 | 195 | 0.033365 | 2.44E-01 | ko00940 |
| Alanine, aspartate and glutamate metabolism | 15 | 48 | 0.045157 | 3.11E-01 | ko00250 |

Supplementary Table S16. The total information of DEGs in W_GA_ vs. W_mock_.

In the individual excel format “Supplementary Table S16”

Supplementary Table S17. KEGG annotation of DEGs in W_GA_ vs. W_mock_.

| Pathway | W_mock_-vs-W_GA_ | All | Pvalue | Qvalue | Pathway ID |
| --- | --- | --- | --- | --- | --- |
| Plant hormone signal transduction | 27 | 214 | 1.42E-10 | 9.36E-09 | ko04075 |
| Pentose and glucuronate interconversions | 14 | 134 | 5.03E-05 | 1.66E-03 | ko00040 |
| Phenylpropanoid biosynthesis | 16 | 195 | 0.000267 | 5.88E-03 | ko00940 |
| Diterpenoid biosynthesis | 4 | 32 | 0.015753 | 2.60E-01 | ko00904 |
| Carotenoid biosynthesis | 4 | 40 | 0.033182 | 4.38E-01 | ko00906 |
| Glutathione metabolism | 7 | 104 | 0.03984 | 4.38E-01 | ko00480 |

Supplementary Table S18. The total information of DEGs in U_GA_ vs. U_mock_.

In the individual excel format “Supplementary Table S18”

Supplementary Table S19. KEGG annotation of DEGs in U_GA_ vs. U_mock_.

| Pathway | U_mock_-vs-U_GA_ | All | P value | Q value | Pathway ID |
| --- | --- | --- | --- | --- | --- |
| Plant hormone signal transduction | 33 | 214 | 1.09E-08 | 1.01E-06 | ko04075 |
| Glutathione metabolism | 20 | 104 | 2.84E-07 | 1.32E-05 | ko00480 |
| ABC transporters | 7 | 32 | 0.000181 | 5.62E-03 | ko02010 |
| Diterpenoid biosynthesis | 6 | 32 | 0.001101 | 2.21E-02 | ko00904 |
| Phenylpropanoid biosynthesis | 21 | 195 | 0.001191 | 2.21E-02 | ko00940 |
| Pentose and glucuronate interconversions | 15 | 134 | 0.004121 | 6.38E-02 | ko00040 |
| Cutin, suberine and wax biosynthesis | 6 | 31 | 0.004803 | 6.38E-02 | ko00073 |
| Tryptophan metabolism | 4 | 27 | 0.011994 | 1.39E-01 | ko00380 |
| Isoquinoline alkaloid biosynthesis | 5 | 31 | 0.021292 | 2.20E-01 | ko00950 |
| Ascorbate and aldarate metabolism | 6 | 44 | 0.026105 | 2.30E-01 | ko00053 |
| Biosynthesis of secondary metabolites | 68 | 1049 | 0.027263 | 2.30E-01 | ko01110 |
| Vitamin B6 metabolism | 3 | 14 | 0.033931 | 2.63E-01 | ko00750 |

Supplementary Table S20. Genome-wide GPI-anchored protein prediction in *P. mume*.

In the individual excel format “Supplementary Table S20”

Supplementary Table S21. DEGs between upright and weeping branches and located on the chromosome 7

| Gene ID | chromosome | start | end | W_ut__fpkm | U_ut__fpkm | log2(FC) | Pvalue | FDR | Symbol | Description |
| --- | --- | --- | --- | --- | --- | --- | --- | --- | --- | --- |
| Pm024032 | Pm7 | 9986862 | 9987188 | 12.42667 | 5.29 | -1.2321 | 0.000266 | 0.019196 | -- | PREDICTED: uncharacterized protein LOC103337641 [Prunus mume] |
| Pm024063 | Pm7 | 10201287 | 10201664 | 0.233333 | 1.8 | 2.947533 | 0.000203 | 0.015988 | -- | PREDICTED: protein SRC1-like [Prunus mume] |
| Pm024165 | Pm7 | 10783468 | 10785803 | 1.51 | 3.766667 | 1.31874 | 9.94E-06 | 0.001493 | NLP6 | PREDICTED: protein NLP6-like isoform X1 [Prunus mume] |
| Pm024167 | Pm7 | 10796671 | 10798904 | 0.663333 | 3.076667 | 2.213562 | 1.05E-07 | 2.81E-05 | SWEET4 | PREDICTED: bidirectional sugar transporter SWEET4-like [Prunus mume] |
| Pm024338 | Pm7 | 11870766 | 11876178 | 0.543333 | 1.903333 | 1.808619 | 1.27E-06 | 0.000245 | At5g50170 | PREDICTED: C2 and GRAM domain-containing protein At5g50170 [Prunus mume] |
| Pm024407 | Pm7 | 12232542 | 12233959 | 2.35 | 8.74 | 1.894973 | 5.72E-06 | 0.000968 | CYSEP | PREDICTED: vignain-like [Prunus mume] |
| Pm024524 | Pm7 | 12846900 | 12848302 | 0.496667 | 3.423333 | 2.785052 | 7.51E-08 | 2.19E-05 | SOC1 | PREDICTED: agamous-like MADS-box protein AGL19 [Prunus mume] |
| Pm024534 | Pm7 | 12903589 | 12905404 | 0.013333 | 0.23 | 4.108524 | 0.000536 | 0.032219 | FATB | PREDICTED: palmitoyl-acyl carrier protein thioesterase, chloroplastic-like isoform X1 [Prunus mume] |
| Pm024536 | Pm7 | 12910507 | 12913635 | 0.303333 | 1.353333 | 2.157541 | 0.000496 | 0.0302 | HSD1 | PREDICTED: 11-beta-hydroxysteroid dehydrogenase 1B-like [Prunus mume] |
| Pm024808 | Pm7 | 14422195 | 14423367 | 1.273333 | 3.53 | 1.471058 | 1.56E-05 | 0.002097 | VDAC1 | PREDICTED: mitochondrial outer membrane protein porin of 36 kDa-like [Prunus mume] |
| Pm025036 | Pm7 | 15673634 | 15675605 | 0.006667 | 0.243333 | 5.189825 | 0.000201 | 0.015952 | CYP78A5 | PREDICTED: cytochrome P450 78A5 [Prunus mume] |
| Pm025050 | Pm7 | 15751873 | 15754328 | 0.63 | 0.093333 | -2.75489 | 2.09E-05 | 0.002567 | GS1-2 | PREDICTED: glutamine synthetase nodule isozyme [Prunus mume] |
| Pm025053 | Pm7 | 15765277 | 15767167 | 0.563333 | 0.03 | -4.23095 | 0.000498 | 0.0302 | -- | PREDICTED: cell division cycle-associated protein 7-like [Ziziphus jujuba] |
| Pm025223 | Pm7 | 16619008 | 16622784 | 10.82333 | 25.53 | 1.238049 | 7.28E-10 | 2.87E-07 | YSL7 | PREDICTED: probable metal-nicotianamine transporter YSL7 [Prunus mume] |

Supplementary Table S22. Primer sequences used for qRT-PCR.

| Gene | Primer Sequences |
| --- | --- |
| *Pm021214(CAD)-F* | TATGCTTCGATGGTACGCCG |
| *Pm021214(CAD)-R* | CCTTGCCAAACTTCACTGCC |
| *Pm023086(PLR)-F* | TCTAGCAGGTCGTTTGCGTT |
| *Pm023086(PLR)-R* | AATGCACTTTGCCCGTCAAC |
| *Pm027000(PE)-F* | TGGCTCAGCTACTTTCACCG |
| *Pm027000(PE)-R* | CTGCGTAGCACCAGATCACA |
| *Pm025897(PG)-F* | GGATGGCAATTCAGCTTCGG |
| *Pm025897(PG)-R* | GCCGAAGTCAGGTCGGTTAT |
| *Pm019059(EXP)-F* | GGATGGCAATTCAGCTTCGG |
| *Pm019059(EXP)-R* | GCCGAAGTCAGGTCGGTTAT |
| *Pm021879(ARG7)-F* | CCAGAAGAAGCGGTTTGTGA |
| *Pm021879(ARG7)-R* | TGTCTTCACTGCAGGGGATTG |
| *Pm021015(SAUR20)-F* | GAGGTTCCAAAAGGGCATGT |
| *Pm021015(SAUR20)-R* | AGCCAAACTCTTCCTCTGCTC |
| *Pm012245(AOP1)-F* | AATACGGCTCTTTCGTGGCA |
| *Pm012245(AOP1)-R* | CCTTACGGTGGCATCCTTGT |
| *Pm024165(NPL6)-F* | TTGCCAGAATGGACCCCAAA |
| *Pm024165(NPL6)-R* | CAGGTTCGTCCAGAGGGTTC |
| *Pm024167(SWEET4)-F* | CTCGTGCTCATGGCTCTTCT |
| *Pm024167(SWEET4)-R* | GACAGCGAGAGGTGAAGCAT |
| *Pm023949(EG)-F* | AACGTGAACTTGGTGGGAGG |
| *Pm023949(EG)-R* | ATAGGCAAGCTGGTTCGTGG |
| *Pm015115(CSL)-F* | GCCTGCTTGAAATCCTGCTC |
| *Pm015115(CSL)-R* | AGCTCAGGAACAGAGCGTAG |
| *Pm029033(PP2A)-F* | AGGGTTCGGCTCGCAATAATAGA |
| *Pm029033(PP2A)-R* | AGCAGCAGCATCACGAATTGAGTAG |
